# Characterizing the top-down sequencing of protein ions prior to mobility separation in a timsTOF†

**DOI:** 10.1039/d2an01682f

**Published:** 2023-03-06

**Authors:** Katherine A. Graham, Charles F. Lawlor, Nicholas B. Borotto

**Affiliations:** a Department of Chemistry, University of Nevada 1664 N. Virginia Street Reno NV 89557 USA nborotto@unr.edu

## Abstract

Mass spectrometry (MS)-based proteomics workflows of intact protein ions have increasingly been utilized to study biological systems. These workflows, however, frequently result in convoluted and difficult to analyze mass spectra. Ion mobility spectrometry (IMS) is a promising tool to overcome these limitations by separating ions by their mass- and size-to-charge ratios. In this work, we further characterize a newly developed method to collisionally dissociate intact protein ions in a trapped ion mobility spectrometry (TIMS) device. Dissociation occurs prior to ion mobility separation and thus, all product ions are distributed throughout the mobility dimension, enabling facile assignment of near isobaric product ions. We demonstrate that collisional activation within a TIMS device is capable of dissociating protein ions up to 66 kDa. We also demonstrate that the ion population size within the TIMS device significantly influences the efficiency of fragmentation. Lastly, we compare CIDtims to the other modes of collisional activation available on the Bruker timsTOF and demonstrate that the mobility resolution in CIDtims enables the annotation of overlapping fragment ions and improves sequence coverage.

## Abstract 2

Here, we demonstrate that protein ions can be dissociated prior to mobility analysis in a trapped ion mobility spectrometry device. Generated product ions are mobility separated enabling facile assignment of near isobaric ions.

## Graphical abstract

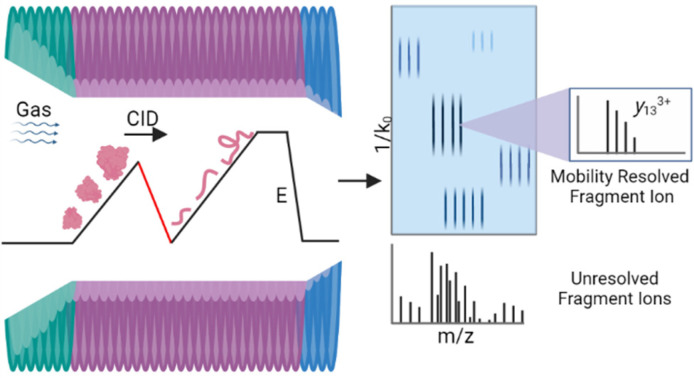

Mass spectrometry (MS)-based proteomics has increasingly focused on the analysis of intact protein ions. These “top-down” analyses enable the annotation of concurrent post-translational modifications and better coverage of highly acidic and hydrophobic regions that are commonly undersampled in bottom-up workflows.^1–6^ While numerous tandem mass spectrometry techniques have been developed for application in top-down workflows, collision-induced dissociation (CID)-techniques remain the most accessible.^7–10^ Collision-based fragmentation, however, typically occurs at the kinetically most labile bonds, shuttling dissociation towards a handful of sites and generating few sequence informative ions.^11–13^ Two-step (MS^3^) collision-based fragmentation is high-throughput and has been shown to increase sequence coverage when applied to protein ions.^14–18^ Despite the promise of these MS^3^ workflows, they often generate overlapping product ions, complicating MS^3^ isolation, and generating convoluted and difficult to analyze mass spectra.^19,20^

Ion mobility spectrometry (IMS) has been employed to overcome the spectral complexity of top-down workflows.^19^ IMS separates ions based on their rotationally-averaged collision cross section, dispersing ions across the mass-to- and size-to-charge dimensions.^17,19,21–24^ If ion dissociation occurs prior to the ion mobility device, IMS can disentangle overlapping isotopic envelopes, facilitating more confident assignments of product ions, and MS^3^ interrogation of previously overlapped product ions, further improving sequence coverage.^19^ While the synergy of IMS and MS^3^-based top-down proteomics could resolve many of these limitations and both Waters traveling wave IMS (TWIMS) and Agilent drift tube equipped instruments with modified sources may be capable of these workflows,^19,25^ this potential still remains largely unexplored.

Trapped ion mobility spectrometry (TIMS) is an IMS variant that with the exception of some custom built systems,^26,27^ was not equipped to dissociate protein ions pre-IMS separation.^22,26,28–31^ To overcome this limitation, we recently developed a collision-based activation technique that achieved dissociation of ubiquitin ions in a commercially available timsTOF.^17^ This dissociation occurs prior to mobility separation and is promoted through careful control of buffer gas pressure and the magnitude of a select DC transfer voltage.^17^ We further demonstrated that these now separated product ions could be further interrogated with the downstream quadrupole and collision cell enabling 50% improved sequence coverage over typical MS^2^ analyses.^17^ While promising, significant questions regarding this pre-IMS activation technique (CIDtims) remain and must be addressed before significant progress towards an MS^3^ workflow can be made.

Here, we further explore the capabilities, limitations, and variables influencing the pre-IMS activation step from this recently developed technique.^17^ We determine that collisional activation within a TIMS device is capable of generating sequence coverage comparable to dedicated collision cells. While sequence coverage decreases with protein mass, we demonstrate the dissociation of protein ions up to 66 kDa. We also reveal that precursor ion count significantly influences product ion abundance and the sequence coverage generated. Lastly, we compare CIDtims to in-source CID and demonstrate that the mobility resolution in CIDtims enables the assignment of previously overlapped product ions and the generation of superior sequence coverage.

## Experimental section

### Materials

Optima LC/MS grade formic acid, methanol, and water were obtained from Fisher Scientific (Waltham, MA). Ubiquitin from bovine erythrocytes, cytochrome C from equine heart, β-lactoglobulin from bovine milk, carbonic anhydrase from bovine erythrocytes, and bovine serum albumin were obtained from Sigma Aldrich (St. Louis, MO).

### Mass spectrometry

Experiments were performed on a Bruker timsTOF (Billerica, MA) trapped ion mobility spectrometry quadrupole-time-of-flight mass spectrometer with an electrospray ionization source. The end plate offset, capillary, and nebulizer were set to 500 V, 4000 V, and 2.0 bar, respectively. Dry gas and dry gas temperature were set to 4 L min^−1^ and 200 °C, respectively. All data was collected with rolling average set to five spectra. Funnel 1, in-source collision induced dissociation (isCID), and deflection delta were set to 320 Vpp, 0 eV, and 70 V, respectively. Funnel 2 and the multipole were both set to 400 Vpp. Ion energy, collision energy, and transfer time were set to 5 eV, 10 eV, and 110 μs, respectively. TIMS experiments were collected with 1/K_0_ scan range, accumulation time, ramp time, and tunnel-in pressure set to 0.5–2 V·s cm^−2^, 20–100 ms, 150 ms, and 2.0–1.35 mbar, respectively. *K*_0_ is the reduced mobility of an ion through a gas and can be utilized to derive the collision cross section (*Ω*) of an ion:
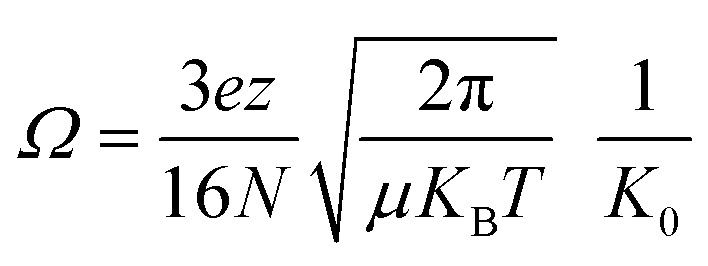
where *K*_B_ is the Boltzman's constant, *T* is temperature, *N* is the buffer gas density, and *μ* is the reduced mass. *z* and *e* are the charge of an ion and elemental charge, respectively. The TIMS DC voltages Δ1–5 were set to 20 V, −120 V, 70 V, 25 V, and 0 V, respectively. The voltage of Δ6 was varied between 30 V and 150 V for ion activation. Samples were diluted in 49/49/2% methanol/water/formic acid to concentrations between 0.5–2.0 μM and injected using direct infusion at a flow rate of 2 μL min^−1^. For CID experiments, precursors were isolated with a width of 5 Da and fragmented with energies between 20 and 75 eV. Spectra were averaged over three minutes.

### Data analysis

CIDtims, isCID, and CID data was analyzed with MASH Explorer search using eTHRASH deconvolution algorithms.^34,35^ Currently, MASH Explorer cannot interpret the mobility dimension of timsTOF data. To overcome this inherent limitation when analyzing CIDtims data, the mobility region was separated into five equally distributed regions of 0.5–0.8, 0.8–1.1, 1.1–1.4, 1.4–1.7, and 1.7–2.1/*K*_0_. Spectra were averaged over each of these mobility windows and extracted with Bruker DataAnalysis 5.2. These spectra were then uploaded into MASH Explorer for analysis (Fig. S1†). Results were confirmed manually for all experiments.

## Results and discussion

**Scheme 1:**
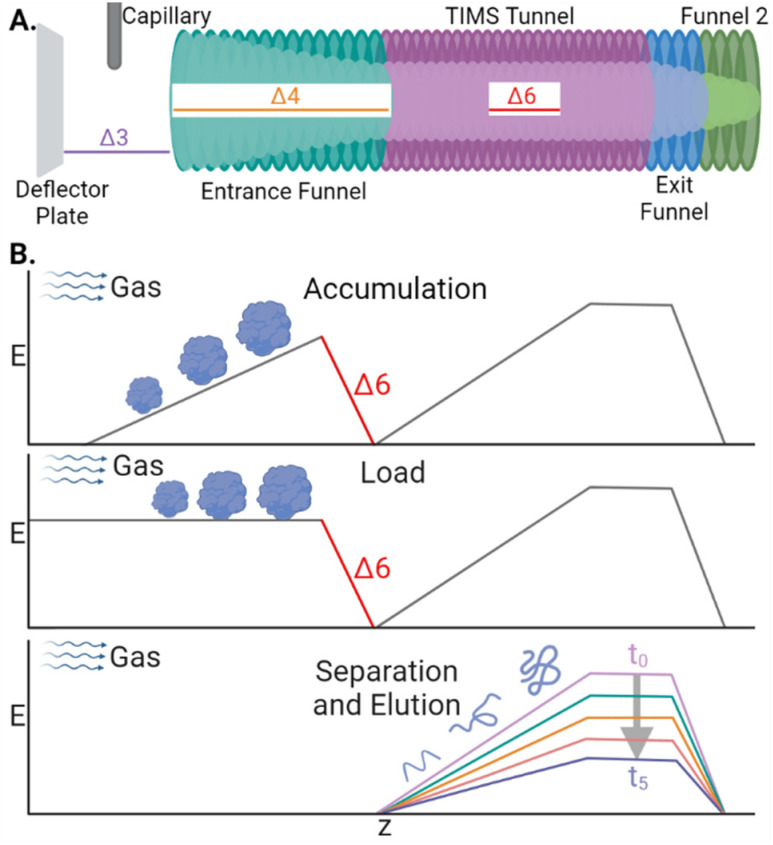
(A) Diagram of the TIMS device with the relevant DC transfer voltages annotated. (B) Electric field plotted during the stages of a TIMS analysis.

CIDtims activation builds off of several studies demonstrating that the internal energy of analytes is correlated with the magnitude of select DC transfer voltages (particularly Δ3, Δ4, and Δ6; Scheme 1A), accumulation time, and is inversely correlated with the TIMS buffer gas pressure (measured as tunnel-in pressure).^29,32,33^ Leveraging these studies, we previously demonstrated that increased Δ6 values were sufficient to dissociate ions of ubiquitin at a tunnel-in pressure of 1.70 mbar.^17^ Ions are subjected to acceleration by the Δ6 voltage as they are transferred from the accumulation region onto the analytical electric field gradient for mobility analysis (Scheme 1B) and thus, any generated product ions are mobility separated. We further characterized the relationship of tunnel-in pressure and Δ6 by examining the collision-induced unfolding of protein ions and found that higher pressures limited the maximum internal energy and degree of protein conformational isomerization.^34^

**Fig. 1:**
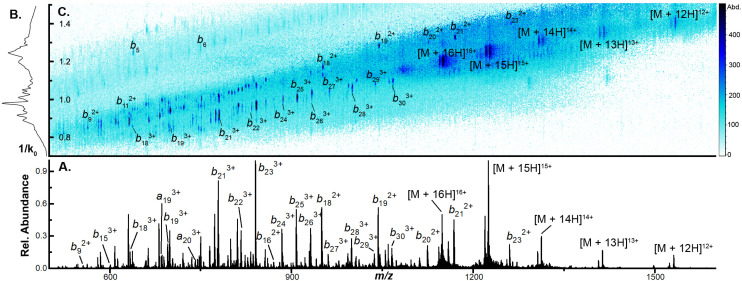
(A) CIDtims mass spectrum of 2 μM β-lactoglobulin (B) mobility spectrum of β-lactoglobulin following CIDtims (C) 2D-IMS-MS plot of dissociated β-lactoglobulin CIDtims with accumulation time of 100 ms and tunnel-in pressure of 1.5 mbar.

To examine the capacity of this activation technique to dissociate incrementally larger protein ions, we initially set the accumulation time to 100 ms, the mobility ramp time to 150 ms (duty cycle ≈ 40%), tunnel-in pressure to 1.5 mbar, Δ6 to 30 V (the minimum value permitted), and directly infused ubiquitin, cytochrome C (CytC), and β-lactoglobulin (βLG). At this low Δ6 setting, no dissociation is observed in any protein ion (Fig. S2A, S3A, and S4A†). When Δ6 is increased to 150 V (the maximum value) the highest charge states of ubiquitin (10+ to 14+), CytC (17+ to 20+), and βLG (16+ to 20+) are fully depleted. This charge state dependence was also observed in our previous work and may reflect dependence on mobile proton-imparted lability as ubiquitin, CytC, and βLG possess 11, 17, and 16 basic sites, respectively. Dissociation of these charge states is sufficient to promote robust product ion formation, generating a sequence coverage of 73 ± 1% (55 ± 1 of 75 amide bonds) for ubiquitin (Fig. S2B†). This sequence coverage represents the dissociation of an additional 29 fragments over that generated in the previous manuscript and is due to the optimization of several TIMS settings and the decreased tunnel-in pressure.^17^ When subjected to CIDtims, CytC and βLG also undergo significant dissociation with 43 ± 2 of 103 (42 ± 2%) and 40 ± 2 of 161 amide bonds (24.6 ± 0.9%) being dissociated, respectively (Fig. 1A and Fig. S3B†). As in our previous work, mobility features corresponding to these generated product ions emerge with increased Δ6 values (Fig. 1B) and can be plotted in a two-dimensional IMS-MS heatmap enabling facile assessment of product ion generation (Fig. 1C and Fig. S2B, S3B†).

### Characterizing the relationship of CIDtims and tunnel pressure

**Fig. 2:**
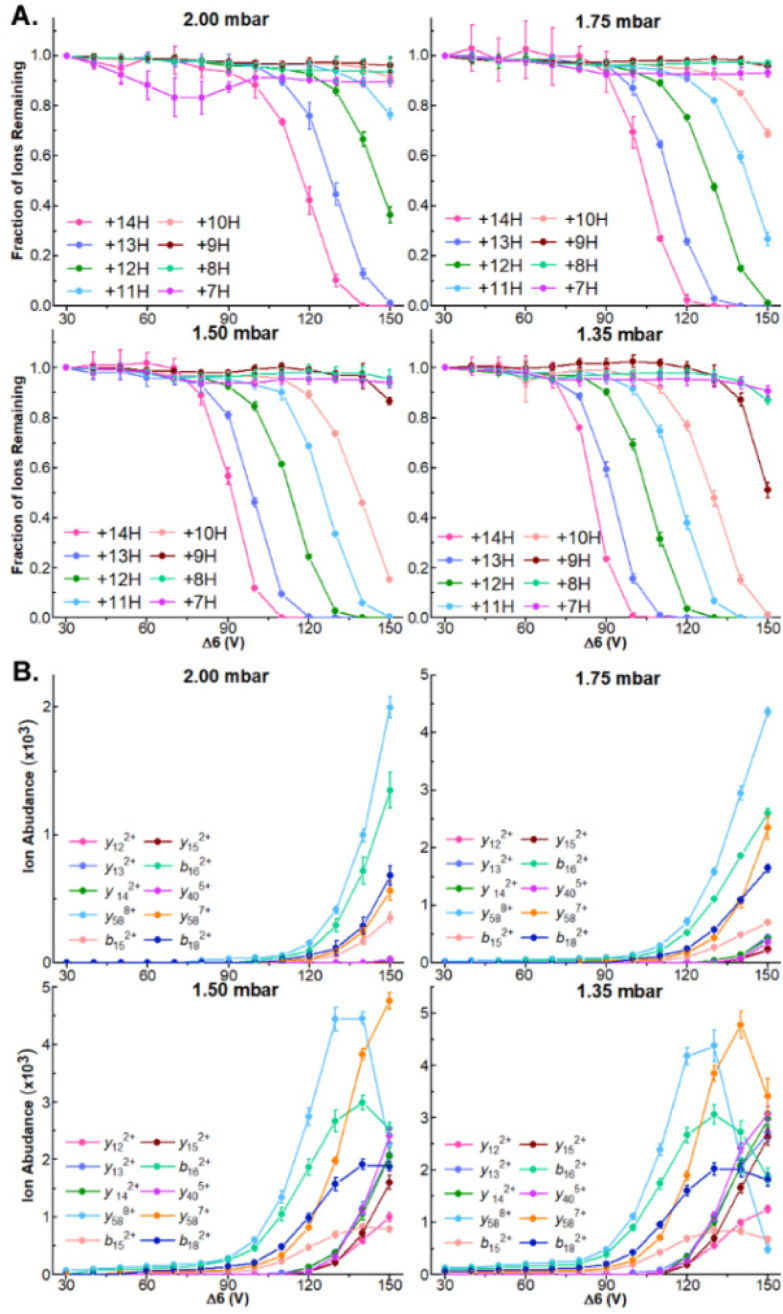
(A) Survival plots of indicated ubiquitin charge states at indicated tunnel-in pressures. (B) Ion abundance of select product ions at indicated Δ6 voltage and tunnel-in pressure.

While robust dissociation and product ion generation is possible at 1.5 mbar, our recent results have found that ion internal energies are inversely correlated with tunnel-in pressure.^17,34^ To systematically examine how this relationship influences the dissociation of protein ions and the generation of sequence coverage across a series of protein ion masses, we directly infused ubiquitin, CytC, and βLG and compared how steadily increasing Δ6 values influences precursor and product ion abundances at tunnel-in pressures of 1.35, 1.5, 1.75, and 2.0 mbar. As in 1.5 mbar, no dissociation is observable in any protein or pressure when Δ6 = 30 V (Fig. 2A and Fig. S5A, S6A†). In fact, no significant precursor ion dissociation occurs until approximately 80 V. At this voltage limited dissociation of the ubiquitin 14+ and 13+ charge states (76.2 ± 0.6% and 88 ± 1% remaining protein ion abundance, respectively) is observable at the lowest tunnel-in pressure tested (1.35 mbar) (Fig. 2A). Incrementally increasing the Δ6 voltage above this value at 1.5 mbar gradually recruits more ubiquitin charge states to dissociate until five charge state are fully depleted at 150 V (Fig. 2A). As demonstrated in our previous manuscripts,^17,34^ increased tunnel-in pressure has an inverse relationship with ion activation and only the 14+ and 13+ charge states of ubiquitin dissociate at 2 mbar even when the highest value of Δ6 is applied (Fig. 2A). Similar trends in ion dissociation are observed for CytC, and βLG as they are subjected to Δ6 activation at various tunnel-in pressures (Fig. S5A and S6A†). To better quantify these trends, we fit each protein charge state dissociation curve with a sigmoidal function and calculated the voltage where 50% of the ion was depleted (Tables S1–S3†). We find that on average the tunnel-in pressures of 1.5, 1.75, and 2 mbar, respectively require 8.2 ± 1.4, 22.8 ± 1.9, and 35 ± 2 higher voltages than those needed at 1.35 mbar to induce similar extents of dissociation.

Δ6-driven depletion of precursor ions also results in robust product ion formation in all proteins (Fig. 2B and Fig. S5B, S6B†). We observe the greatest product ion abundance when Δ6 = 150 V and will utilize that value for all future experiments. When ubiquitin is subjected to CIDtims at 2.0, 1.75, 1.5, and 1.35 mbar, 42.7 ± 0.6, 48 ± 1, 55 ± 1, and 55 ± 2 of the 75 amide bonds dissociate, respectively. This behavior of increasing sequence coverage as tunnel-in pressure is decreased continues for CytC and βLG with 30 ± 2, 36.7 ± 0.6 and 43 ± 2 bonds dissociating out of 103 for CytC, (Fig. S3B†) and 26 ± 2, 35 ± 2, and 40 ± 2 of 161 amide bonds dissociating for βLG at tunnel in pressures of 2.0, 1.75, and 1.5 mbar, respectively (Fig. S4B†). At 1.35 mbar operation of the TIMS device becomes challenging for these proteins, and thus, we were unable to reliably measure these values. Lastly, reduction of tunnel-in pressure is known to decrease the resolution of the mobility measurement.^35^ Despite this decrease in resolution, we see no reduction in our ability to mobility resolve and assign product ions in any protein. In fact, we see a steady increase in sequence coverage as pressure decreases (Fig. S7†), likely stemming from the increased activation energy available at these lower pressures. Due to 1.5 mbar generating the most reproducibly high sequence coverage it will be used for all following experiments.

### Dissociation of large protein ions in the TIMS device

**Fig. 3:**
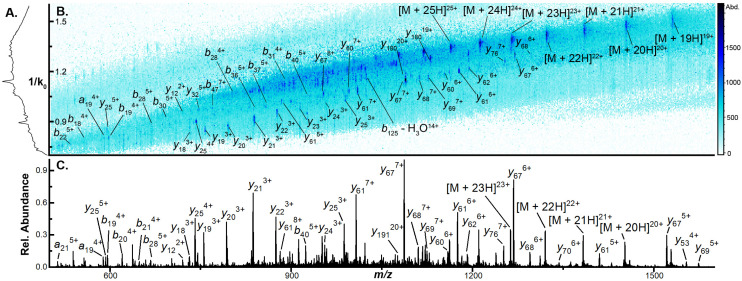
(A) CIDtims mass spectrum of 0.5 μM carbonic anhydrase (B) mobility spectrum of carbonic anhydrase following CIDtims (C) 2D-IMS-MS plot of dissociated carbonic anhydrase with accumulation time of 20 ms and tunnel-in pressure of 1.5 mbar.

**Fig. 4:**
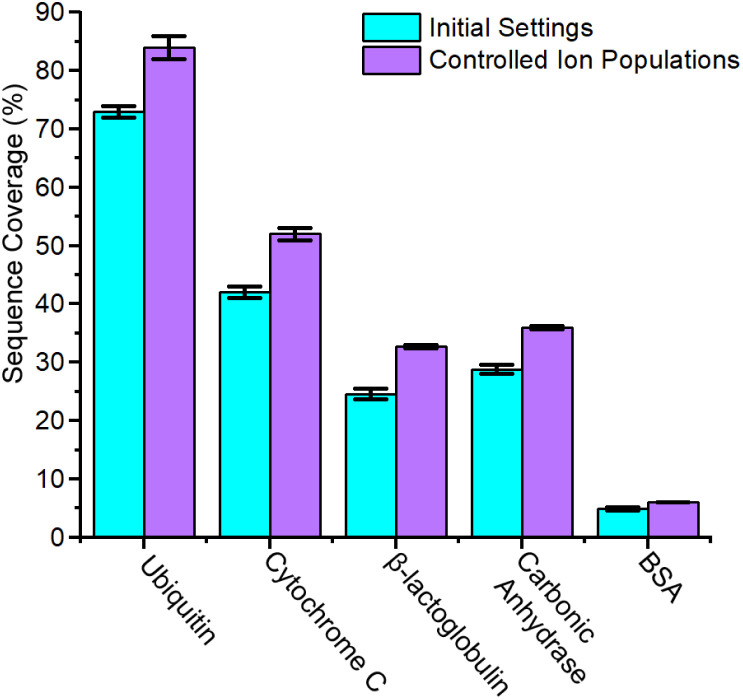
Sequence coverage for proteins at initial settings and updated settings with controlled ion populations. Ion populations were reduced *via* decreased accumulation time for all proteins. In addition to these altered accumulation times, the sample concentration for BSA and carbonic anhydrase were also decreased.

The capacity to generate and annotate a diverse set of sequence informative ions increases in difficulty as the mass of the interrogated protein ion rises.^36–38^ To evaluate the applicability of CIDtims to large protein ions, we directly infused carbonic anhydrase (29 kDa) and bovine serum albumin (BSA) (66 kDa) and subjected each to CIDtims. Unlike ubiquitin, CytC, and βLG, however, when carbonic anhydrase is analyzed at Δ6 values of 30 V significant fragmentation is observed (Fig. S8†). Reduction of the accumulation time within the TIMS device decreases the observed activation (Fig. S8†) and at 20 ms, carbonic anhydrase remains intact. When Δ6 is increased to 150 V the highest charge states of carbonic anhydrase (45+ to 26+) are fully depleted. This dissociation promotes the fragmentation of 74 ± 2 of the 258 amide bonds and the generation of 28.8 ± 0.8% sequence coverage (Fig. S9†). When the even larger BSA is interrogated at these settings, 29 ± 2 of 582 amide bonds are fragmented resulting in 4.9 ± 0.3% sequence coverage (Fig. S10†). While fragmentation of BSA ions is observed, the signal-to-noise ratios of the remaining intact ions is poor. The TIMS device has been shown to be susceptible to space-charge effects and space-charge can result in the preferential loss of high-*m*/*z* ions and ion activation.^33,39–44^ To assess if TIMS overfilling and space charge effects are the cause of these observations, we incrementally decreased the concentration of carbonic anhydrase from 2 μM to 0.5 μM. As the concentration of this protein and subsequently the abundance of ions associated with it are decreased, the relative abundance of high *m*/*z* ions increases (Fig. S11†) signifying that these ions were indeed selectively suppressed likely due to space charge. When the lowest concentration of carbonic anhydrase is subjected to CIDtims fragmentation, the increased stability of high *m*/*z* ions also applies to the generated product ions and the annotation of an additional 19 product ions is possible. Including these additional product ions, CIDtims dissociates 93 ± 1 of the 258 amide bonds (36.0 ± 0.3% sequence coverage) in carbonic anhydrase (Fig. 3, 4 and Fig. S12†).

**Fig. 5:**
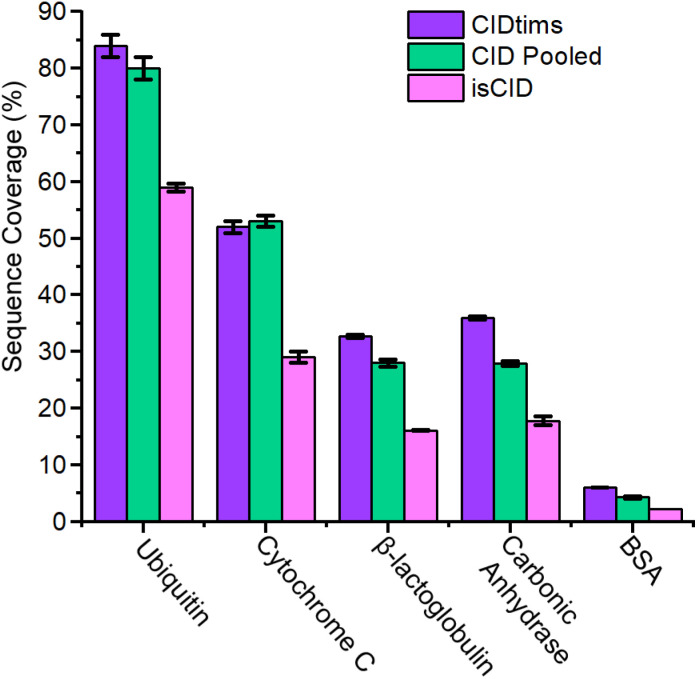
Sequence coverage of CIDtims, pooled CID of ubiquitin 12+ to 14+, CytC 14+, 15+ and 8+, BLG 13+ to 15+, carbonic anhydrase 33+ to 31+, BSA 48+ to 50+, and in-source CID.

While space charge effects were not immediately obvious in the prior analyses of CytC and βLG, reduction of accumulation time to 20 ms alone is sufficient to dramatic increase the abundance of the lowest charge states of each protein ion (Fig. S13 and S14†). CIDtims of these two protein ions again generates significantly more high-*m*/*z* product ions increasing sequencing coverage by 24.9, and 33% for CytC and βLG, respectively (Fig. S15 and S16†). This leads to a total sequence coverage of 52 ± 1% for CytC and 32.7 ± 0.3% for βLG. While we anticipate sequence coverage to decrease with increasing mass, the dissociation of βLG generates lower sequence coverage than the dissociation of carbonic anhydrase (Fig. 5). This decreased sequence coverage is due to the presence of two disulfide bonds in the protein structure and the generation of few sequence informative ions between the participating cysteine residues (Fig. S16†). We will explore the use of reducing agents at improving the sequence coverages of this protein and BSA which has 17 disulfide bonds in future work. While the mass spectrum of intact ubiquitin only demonstrates minor changes upon the reduction of accumulation time (Fig. S17†), when subjected to CIDtims, 63 ± 2 of 75 amide bonds (84 ± 2% sequence coverage) were dissociated (Fig. S18†). The sequence coverages generated with this technique are comparable to recent work completed on a custom built tandem-TIMS (tTIMS) instrument which produced sequence coverages of 88%, 42%, and 32% for ubiquitin, CytC, and βLG, respectively.^22^

When 0.5 μM BSA is interrogated with a 20 ms accumulation time, no increase in sequence coverage is observed indicating that these alterations in accumulation time and concentration are insufficient to reduce space-charge effects (Fig. S19†). The reduced signal-to-noise ratio of the remaining intact protein ions, however, still suggests that suppression may be occurring. To alleviate this, we utilized the ion charge control (ICC) setting which enables the dynamic control of accumulation time and the number of charges present within the TIMS device. Limiting the number of charges to 3.5 million is successful at reducing space charge and results in the addition of six product ions and dissociation of 35.3 ± 0.6 of 582 amide bonds and 6.07 ± 0.09% sequence coverage (Fig. S20†). On average, reducing the ion count in the TIMS device resulted in a ≈24.1% improvement in sequence coverage for the interrogated protein ions (Fig. 4). The dramatic influence of space charge on sequence coverage generation indicates that the dynamic control of accumulation time will be necessary for this technique to be employed to complex mixtures of proteins with highly variable concentrations. In future work, we will further examine the utility of ICC at limiting the influence of space charge in the TIMS device.^45^

### Comparison of CIDtims to other modes of collisional activation available on the timsTOF

To assess how effective CIDtims could be for the analysis of intact protein ions, we fragmented each of these proteins with in-source CID (isCID) and conventional CID. Both isCID and conventional CID occur downstream of the TIMS device and thus, product ions will not be ion mobility separated. isCID is implemented in funnel 2 (Scheme 1) and the collision cell is employed for conventional CID. When ubiquitin, CytC, βLG, carbonic anhydrase, and BSA are activated with isCID it demonstrates a similar charge state dependence as CIDtims with only the highest charge states for each protein dissociating (Fig. S21–S25†). Dissociation of these charge states results in robust product ion generation with 44.3 ± 0.6 of 75 (59.1 ± 0.7%), 30 ± 1 of 103 (29.1 ± 0.9%), 26 ± 2 of 161 (16 ± 1%), 46 ± 2 of 258 (17.8 ± 0.8%), and 13 ± 0 of 582 amide bonds (2.2 ± 0%) dissociated for ubiquitin, CytC, βLG, carbonic anhydrase, and BSA, respectively (Fig. 5 and Fig. S21–S25†). This is notably lower sequence coverage than that generated by CIDtims (Fig. 5).

**Fig. 6:**
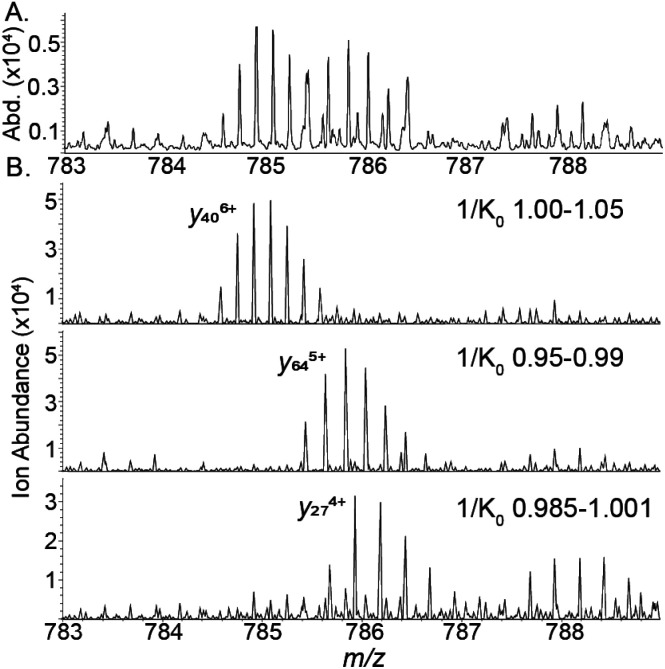
(A) isCID fragment ions of cytochrome C (B) CIDtims mobility resolved product ions of cytochrome C.

The increased performance of CIDtims is partially attributable to both, IMS-derived improvements in signal-to-noise ratios and disentanglement of overlapping product ions. This is exemplified when isCID is applied to ions of CytC. Dissociation of this protein generates a convoluted mixture of overlapping isotopic patterns (Fig. 6A) prohibiting the confident assignment of any product ions. When CIDtims is employed, however, the mobility separation enables facile assignment of the *y*_27_^4+^, *y*_40_^6+^, and *y*_34_^5+^ product ions (Fig. 6B). The mobility separation also facilitates the isolation and interrogation of these ions with the downstream quadrupole and collision cell making CIDtims an ideal first step in future pseudo-MS^3^ workflows.

When the dedicated collisional cell is utilized to dissociate individual charge states, only moderate sequence coverage is obtained. Pooling the generated product ions from the dissociation of multiple charge states as recommended by Mcluckey *et al.*,^11^ however, increases the sequence coverage generated. Pooling the generated product ions from the three most abundant charge states of ubiquitin (12+, 11+, and 10+) results in the dissociation of 60 ± 2 out of 76 amide bonds and 80 ± 2% sequence coverage a 7% percent improvement over a single charge state. (Fig. S26 and S27A†). Cytochrome C, βLG, carbonic anhydrase, and BSA see a similar improvement, each demonstrating sequences coverages of 53 ± 1%, 27.9 ± 0.6%, 27.9 ± 0.4%, and 4.4 ± 0.1% and improvements of 10%, 8%, 16%, and 33%, respectively when their three most abundant charge states are pooled (Fig. S27B–E, S28–S31†). CIDtims generates superior sequence coverage than CID for ubiquitin, βLG, carbonic anhydrase, and BSA by 4.9%, 17%, 29.2%, and 39.5%, respectively. CID outperforms CIDtims for CytC by 1.2%. The increase in sequence coverage for CytC is due to the fragmentation of the 8+ charge state generating unique fragment ions that are not found in the other interrogated charge states. Overall, CIDtims generates comparable data to CID all while still retaining the potential to be utilized in an MS^3^ workflow.

## Conclusions

In summary, we have used CIDtims to fragment intact protein ions of ubiquitin, cytochrome C, β-lactoglobulin, carbonic anhydrase, and bovine serum albumin. We further characterized the relationship between tunnel-in pressure and the Δ6 voltage on the dissociation of protein ions. Furthermore, we determined that minimizing space-charge effects in the device results in a significant increase in sequence coverage for all proteins examined. When compared to the other modes of collisional activation on a timsTOF, CIDtims generates comparable sequence coverage to CID of three most abundant charge states combined. While the simultaneous activation of all charge states results in complex mass spectra, the additional mobility dimension separates overlapping product ions and results in higher sequence coverage than isCID. When coupled with activation in the ensuing collision cell and liquid chromatographic separation, we envision this collisional activation technique as an integral component of a powerful pseudo-MS^3^ workflow that can effectively sequence complex mixtures of intact proteins.

## Author contributions

The manuscript was written through contributions of all authors. All authors have given final approval to the final version of the manuscript.

## Conflicts of interest

There are no conflicts to declare.

## Supplementary Material

AN-148-D2AN01682F-s001
