# Supplementary material for: Characterizing the top-down sequencing of protein ions prior to mobility separation in a timsTOF
Source: Analyst. 2023 Mar 6;148(7):1534–42. doi: 10.1039/d2an01682f (PMC10042122; doi:10.1039/d2an01682f)

Electronic Supplementary Material (ESI) for Analyst.
This journal is © The Royal Society of Chemistry 2023

**Supporting Information**

**Characterizing the Top-down Sequencing of Protein Ions Prior to Mobility Separation in a timsTOF**

Katherine A. Graham, Charles F. Lawlor, and Nicholas B. Borotto*

Department of Chemistry, University of Nevada, 1664 N. Virginia Street Reno, NV 89557, USA

**Contents**

Figure S1. CIDtims Analysis Workflow ....... 2
Figure S2. Ubiquitin mass spectrum, CIDtims spectrum, mobility spectrum, and 2D-IMS-MS ....... 3
Figure S3. Cytochrome C mass spectrum, CIDtims spectrum, mobility spectrum, and 2D-IMS-MS ....... 4
Figure S4. β-lactoglobulin mass spectrum, mobility spectrum, and 2D-IMS-MS ....... 5
Figure S5. β-lactoglobulin Survival Plots ....... 6
Figure S6. Cytochrome C Survival Plots ....... 7
Table S1. Voltage and pressure values where 50% of indicated ubiquitin charge state is dissociated. ...7
Table S2. Voltage and pressure values where 50% of indicated Cytochrome C charge state is dissociated. ....... 8
Table S3. Voltage and pressure values where 50% of indicated β-lactoglobulin charge state is dissociated. ....... 8
Figure S7. Influence of tunnel-pressure on sequence coverage generation ....... 8
Figure S8. Spectra of carbonic anhydrase at decreasing accumulation times ....... 9
Figure S9. Carbonic anhydrase CIDtims spectrum, mobility spectrum, and 2D-IMS-MS ....... 10
Figure S10. BSA CIDtims spectrum, mobility spectrum, and 2D-IMS-MS ....... 11
Figure S11. Carbonic anhydrase mass spectra at various concentrations ....... 12
Figure S12. Carbonic anhydrase CIDtims fragmentation ladder ....... 13
Figure S13. Mass spectra of intact cytochrome C at indicated accumulation times ....... 14
Figure S14. Mass spectra of intact β-lactoglobulin at indicated accumulation times ....... 14
Figure S15. Cytochrome C CIDtims spectrum, mobility spectrum, and 2D-IMS-MS with reduced accumulation ....... 15
Figure S16. β-lactoglobulin CIDtims spectrum, mobility spectrum, and 2D-IMS-MS with reduced accumulation ....... 16
Figure S17. Mass spectra of intact Ubiquitin at varied accumulation times ....... 16
Figure S18. Ubiquitin CIDtims mass spectrum, mobility spectrum, and 2D-IMS-MS with reduced accumulation ....... 17
Figure S19. BSA CIDtims spectrum, mobility spectrum, and 2D-IMS-MS at 0.5 μM ....... 18
Figure S20. BSA CIDtims spectrum, mobility spectrum, and 2D-IMS-MS with ICC ....... 19
Figure S21. Ubiquitin isCID mass spectrum and ladder ....... 20
Figure S22. Cytochrome C isCID mass spectrum and ladder ....... 20
Figure S23. β-lactoglobulin isCID mass spectrum and ladder ....... 21
Figure S24. Carbonic Anhydrase isCID mass spectrum and ladder ....... 22
Figure S25. BSA isCID mass spectrum and ladder ....... 23
Figure S26. Ubiquitin CID spectrum and ladder ....... 24
Figure S27. Venn diagram of CID-generated product ions for indicated charge states. ....... 24
Figure S28. Tandem mass spectrum of cytochrome C following CID ....... 25
Figure S29. Tandem mass spectrum of β-lactoglobulin following CID ....... 25
Figure S30. Tandem mass spectrum of Carbonic anhydrase following CID ....... 26

Figure S31. Tandem mass spectrum of BSA following CID ....................................................................27

**Figure S1. CIDtims Analysis Workflow**
MASH workflow for CIDtims data. **A.** CIDtims tandem mass spectrum of ubiquitin. **B.** Highlighted regions indicate the mobility bins which are averaged and exported. **C.** Mobility resolved mass spectra of indicated mobility bin. Fragment ions labeled in blue are identified only in the mobility resolved spectra.

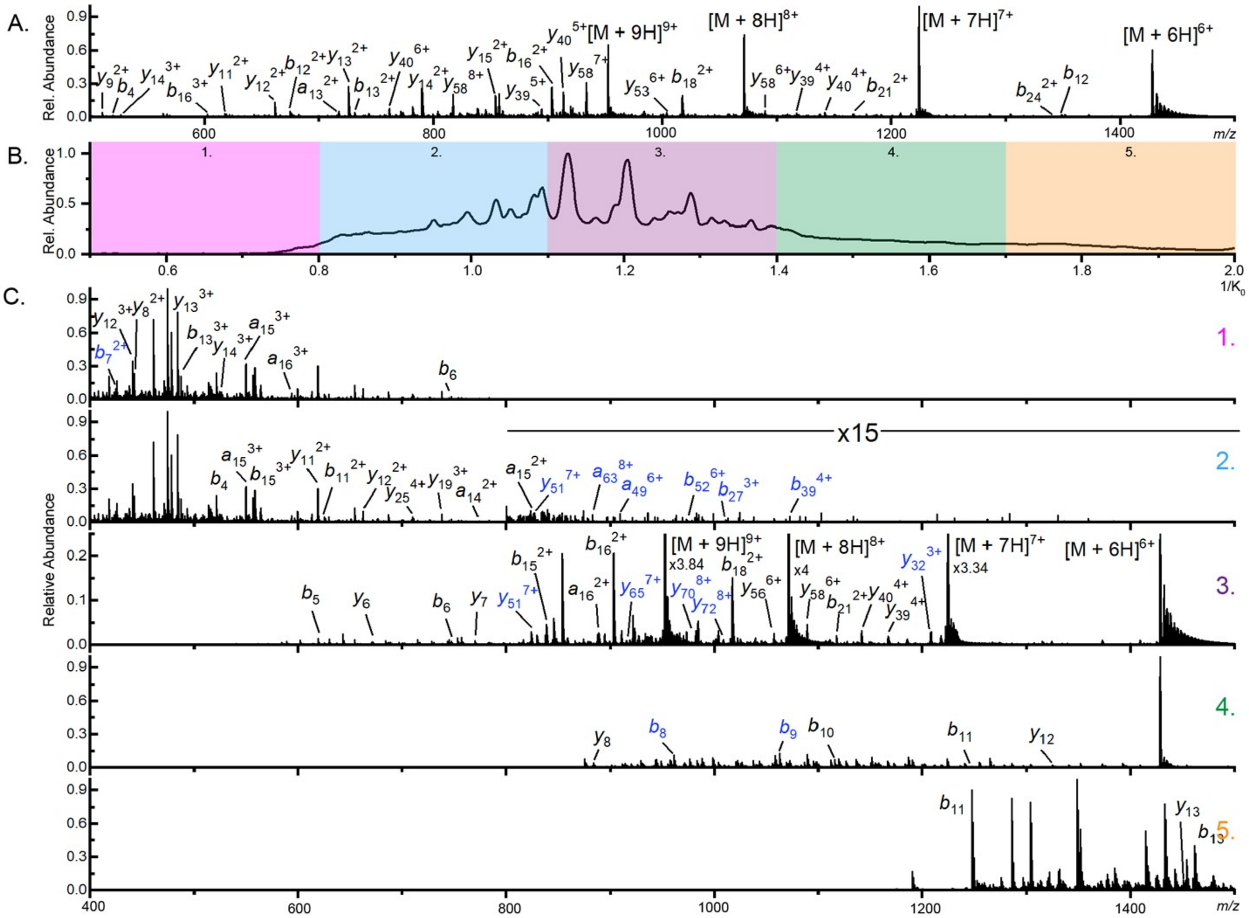

**Figure S2. Ubiquitin mass spectrum, CIDtims spectrum, mobility spectrum, and 2D-IMS-MS**

Mass spectrum, mobility spectrum, and 2D-IMS-MS plot of 2 μM ubiquitin generated by CIDtims when tunnel-in pressure is 1.5 mbar, accumulation time is 100 ms, and Δ6 is **A.** 30 V and **B.** 150 V with ladder of resulting fragment ions. Fragments in purple are identified only from the mobility resolved spectra.

**A.**

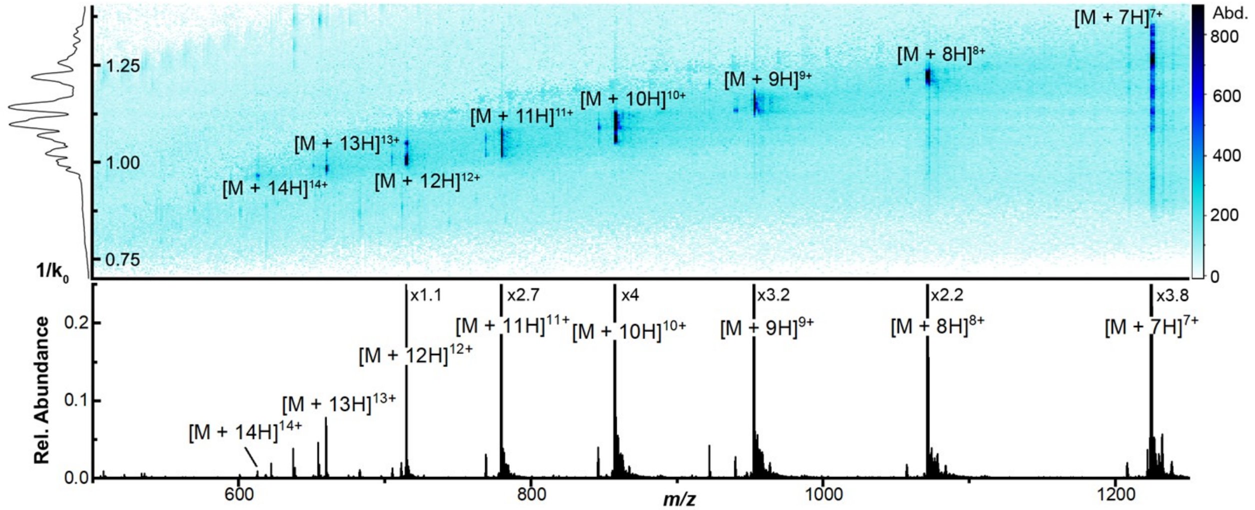


**B.**

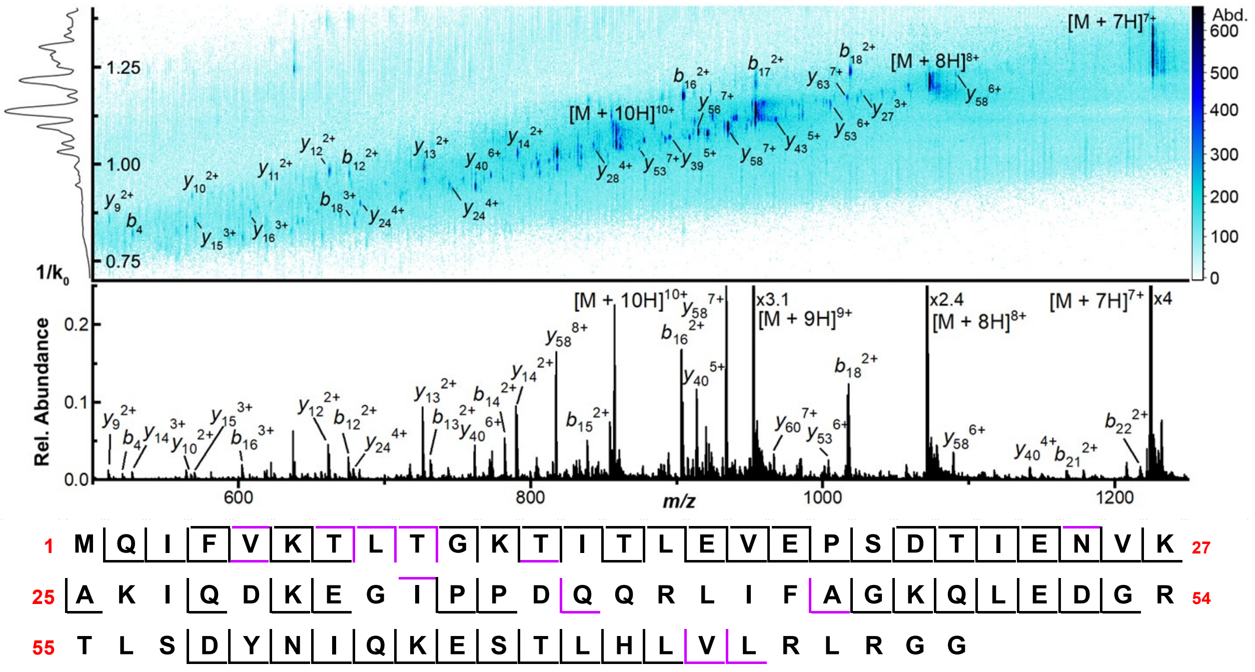

**Figure S3. Cytochrome C mass spectrum, CIDtims spectrum, mobility spectrum, and 2D-IMS-MS**

Mass spectrum, mobility spectrum, and 2D-IMS-MS plot of 2 μM cytochrome C generated by CIDtims when tunnel-in pressure is 1.5 mbar, accumulation time is 100 ms, and Δ6 is **A.** 30 V and **B.** 150 V with ladder of resulting fragment ions. Fragments in purple are identified only from the mobility resolved spectra.

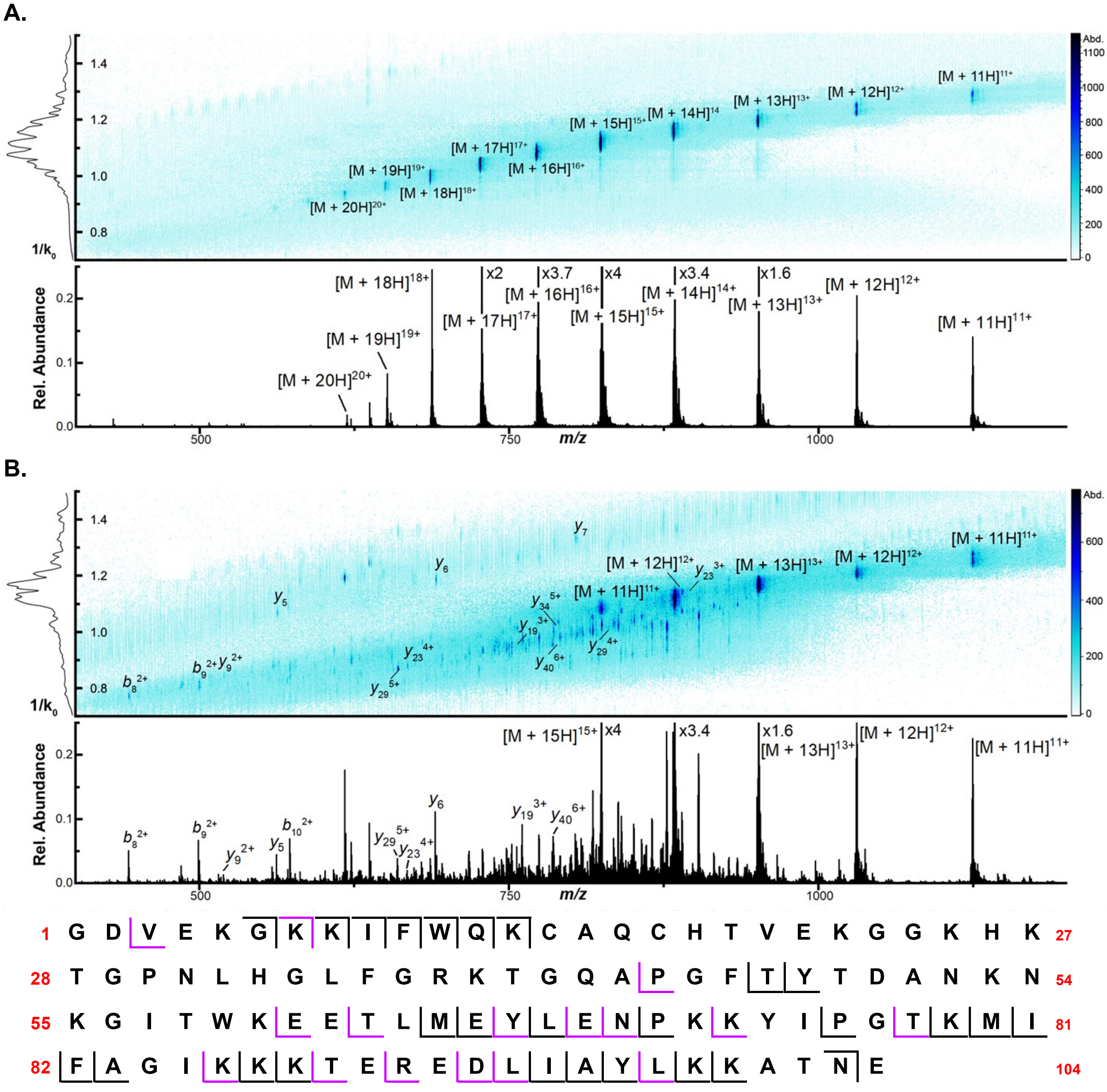

**Figure S4. β-lactoglobulin mass spectrum, mobility spectrum, and 2D-IMS-MS**

Mass spectrum, mobility spectrum, and 2D-IMS-MS plot of 2 μM β-lactoglobulin generated by CIDtims when tunnel-in pressure is 1.5 mbar, accumulation time is 100 ms, and Δ6 is **A.** 30 V and **B.** 150 V with ladder of resulting fragment ions. Fragments in purple are identified only from the mobility resolved spectra.

**A.**

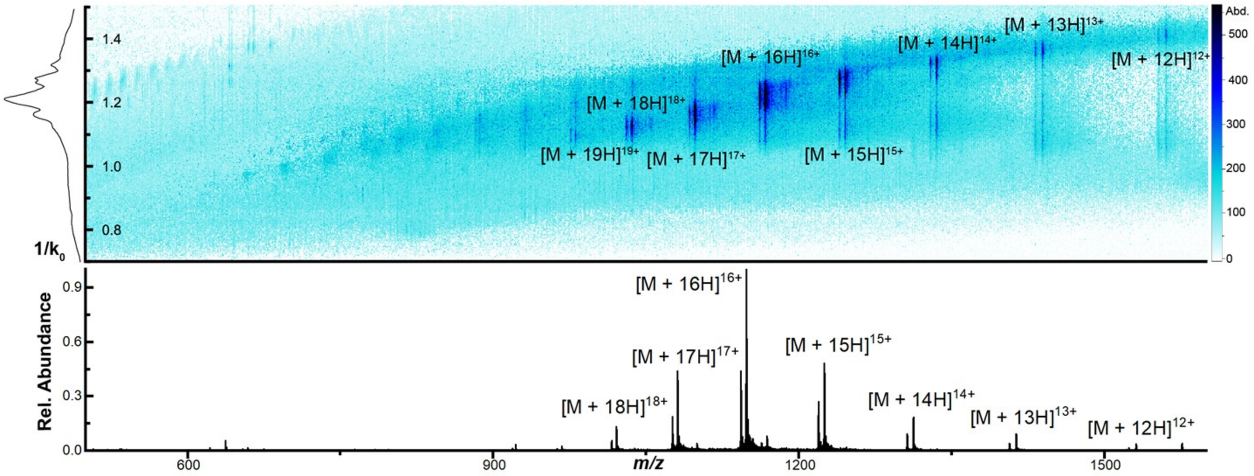


**B.**

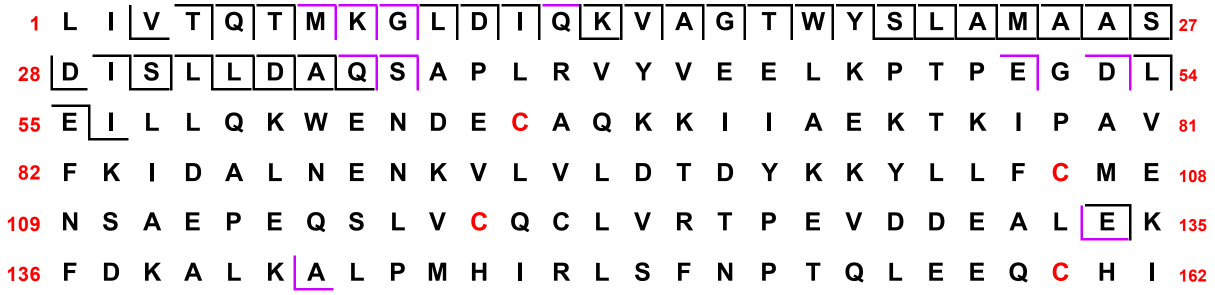

**Figure S5. β-lactoglobulin Survival Plots**

**A.** Survival plots of indicated β-lactoglobulin charge states at indicated tunnel-in pressures. **B.** Ion abundance of select product ions at indicated ∆6 voltage and tunnel-in pressures.

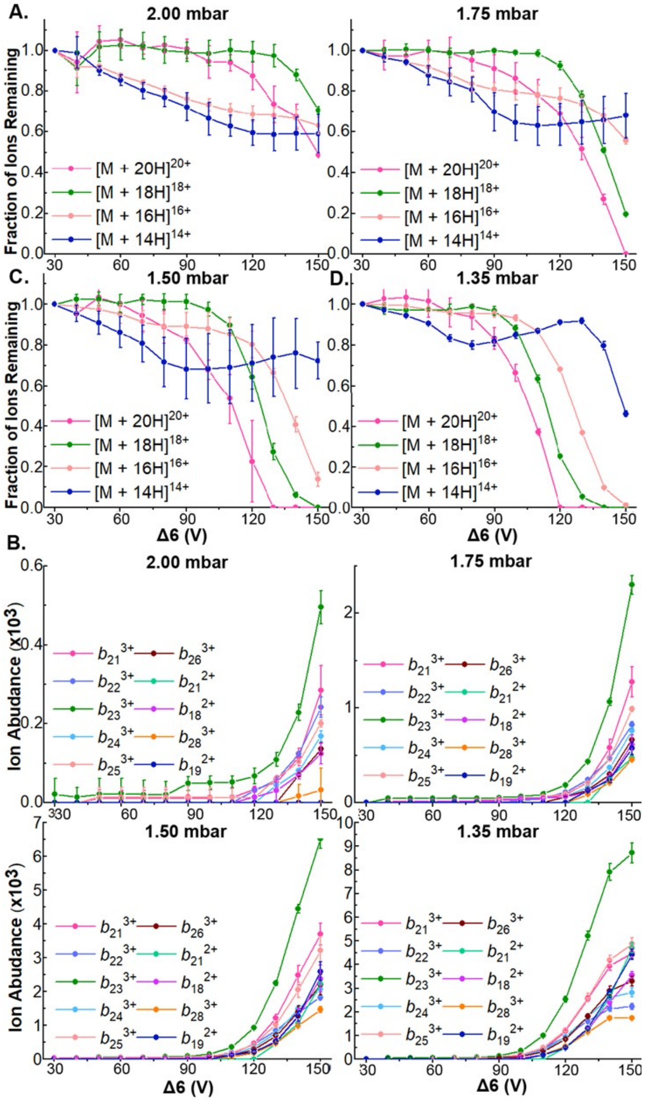

**Figure S6. Cytochrome C Survival Plots**

**A.** Survival plots of indicated cytochrome C charge states at indicated tunnel-in pressures. **B.** Ion abundance of select product ions at indicated ∆6 voltage and tunnel-in pressures.

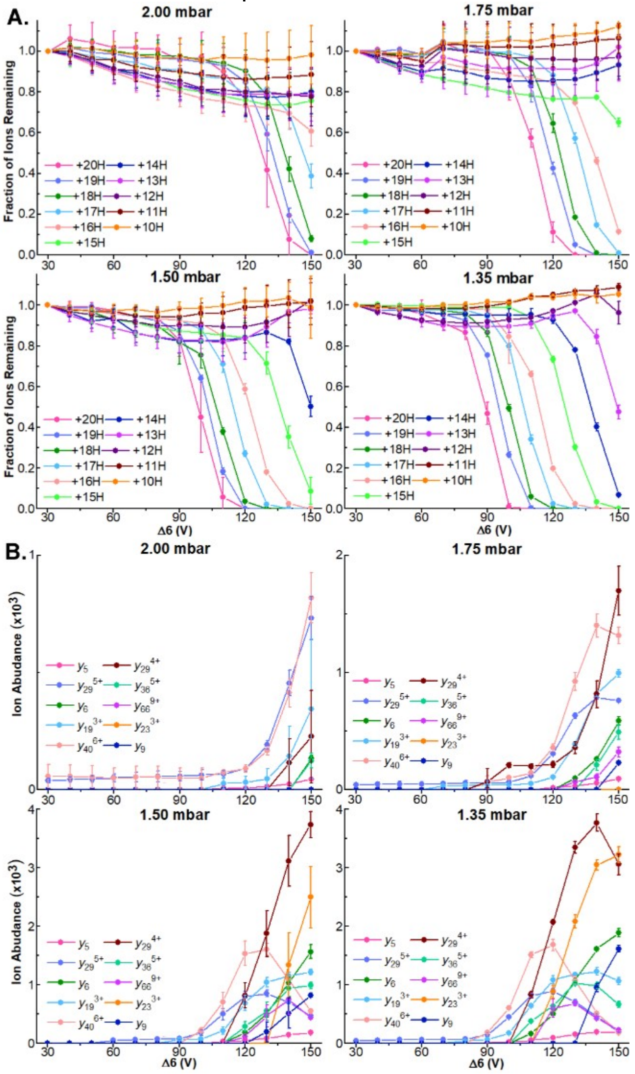


**Table S1. Voltage and pressure values where 50% of indicated ubiquitin charge state is dissociated.**

The voltage and pressure where 50% of the indicated ubiquitin charge state is dissociated. Value was calculated by fitting each survival curve to a Boltzmann sigmoidal function.

| Charge State | Pressure | | | |
|---|---|---|---|---|
| | 2.0 mbar | 1.75 mbar | 1.5 mbar | 1.35 mbar |
| $[M+14H]^{14+}$ | 117 V | 104 V | 91 V | 85 V |
| $[M+13H]^{13+}$ | 128 V | 114 V | 99 V | 92 V |
| $[M+12H]^{12+}$ | 139 V | 129 V | 113 V | 105 V |
| $[M+11H]^{11+}$ | N/A | 138 V | 125 V | 117 V |
| $[M+10H]^{10+}$ | N/A | N/A | 136 V | 129 V |
| $[M+9H]^{9+}$ | N/A | N/A | N/A | 143 V |

**Table S2. Voltage and pressure values where 50% of indicated Cytochrome C charge state is dissociated.**

The voltage and pressure where 50% of the indicated cytochrome C charge state is dissociated. Value was calculated by fitting each survival curve to a Boltzmann sigmoidal function.

| Charge State | Pressure | | | |
|---|---|---|---|---|
| | 2.0 mbar | 1.75 mbar | 1.5 mbar | 1.35 mbar |
| $[M+20H]^{20+}$ | 127 V | 111 V | 99 V | 89 V |
| $[M+19H]^{19+}$ | 132 V | 118 V | 103 V | 95 V |
| $[M+18H]^{18+}$ | 136 V | 123 V | 107 V | 100 V |
| $[M+17H]^{17+}$ | 140 V | 131 V | 115 V | 106 V |
| $[M+16H]^{16+}$ | N/A | 137 V | 122 V | 113 V |
| $[M+15H]^{15+}$ | N/A | N/A | 135 V | 125 V |
| $[M+14H]^{14+}$ | N/A | N/A | N/A | 137 V |

**Table S3. Voltage and pressure values where 50% of indicated β-lactoglobulin charge state is dissociated.**

The voltage and pressure where 50% of the indicated β-lactoglobulin charge state is dissociated. Value was calculated by fitting each survival curve to a Boltzmann sigmoidal function.

| Charge State | Pressure | | | |
|---|---|---|---|---|
| | 2.0 mbar | 1.75 mbar | 1.5 mbar | 1.35 mbar |
| $[M+20H]^{20+}$ | N/A | 131 V | 111 V | 105 V |
| $[M+19H]^{19+}$ | N/A | 132 V | 119 V | 110 V |
| $[M+18H]^{18+}$ | N/A | 137 V | 124 V | 114 V |
| $[M+17H]^{17+}$ | N/A | N/A | 129 V | 119 V |
| $[M+16H]^{16+}$ | N/A | N/A | 134 V | 126 V |
| $[M+15H]^{15+}$ | N/A | N/A | N/A | 132 V |

**Figure S7. Influence of tunnel-pressure on sequence coverage generation**

Plotted sequence coverage of ubiquitin, cytochrome C, and β-lactoglobulin generated by CIDtims at tunnel-in pressures of 2.0 mbar, 1.75 mbar, and 1.5 mbar.

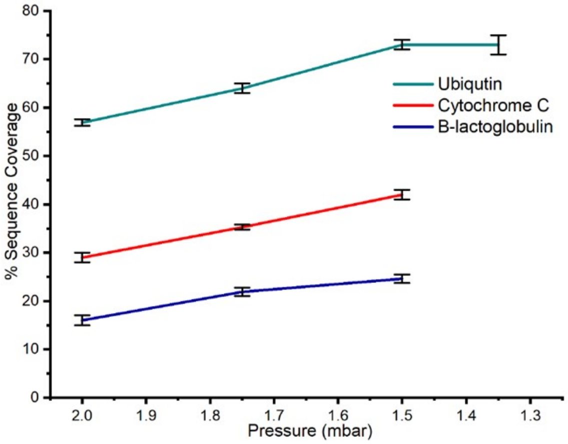

**Figure S8. Spectra of carbonic anhydrase at decreasing accumulation times**

Mass spectra of 2.0 uM carbonic anhydrase as accumulation time is incrementally decreased. tunnel-in pressure was set to 1.5 mbar and Δ6 was set to 30 V.

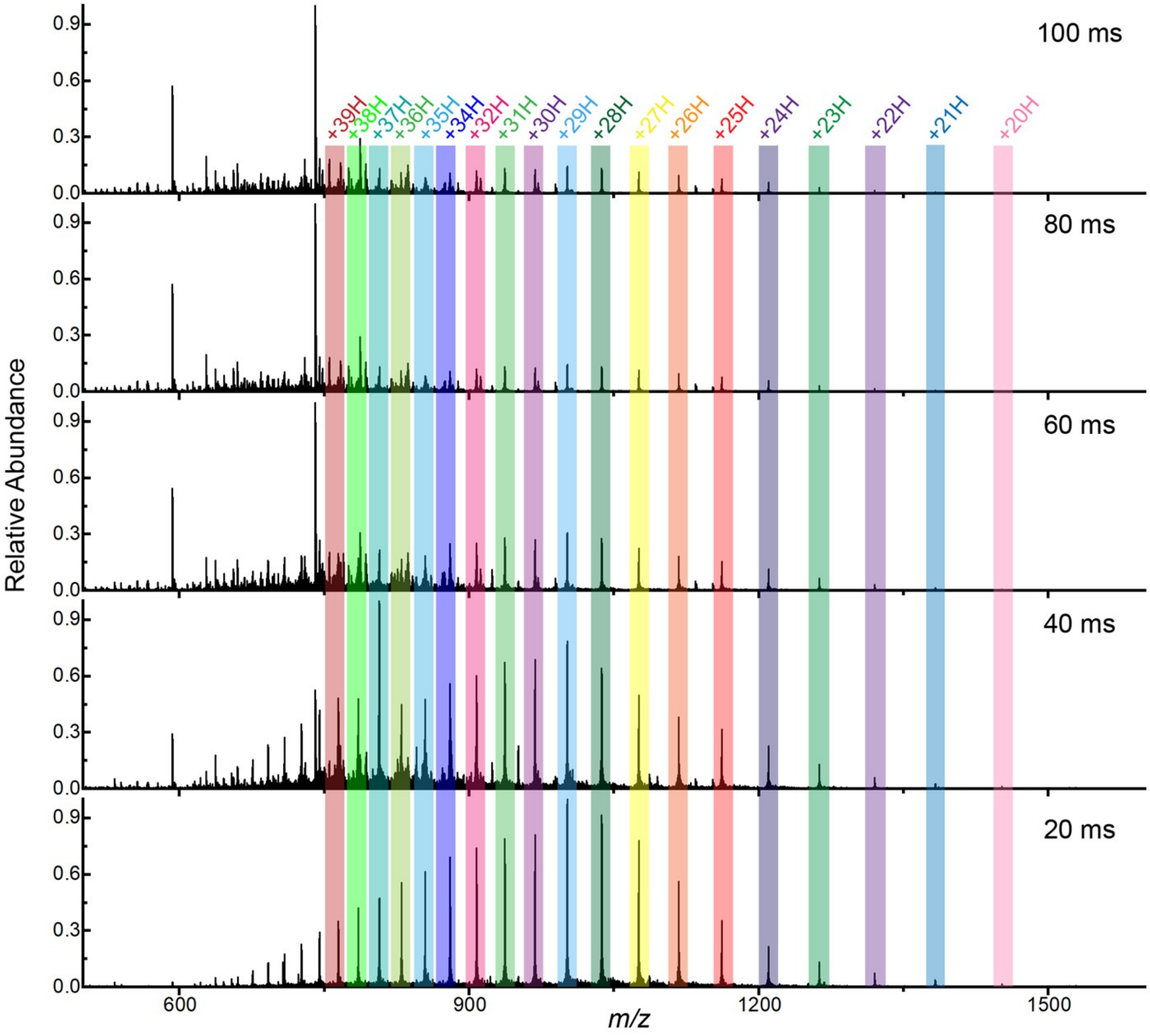

**Figure S9. Carbonic anhydrase CIDtims spectrum, mobility spectrum, and 2D-IMS-MS**

Mass spectrum, mobility spectrum, and 2D-IMS-MS plot of 2 μM carbonic anhydrase generated by CIDtims when tunnel-in pressure is 1.5 mbar, accumulation time is 20 ms, and Δ6 is 150 V with ladder of resulting fragment ions. Fragments in purple are identified only from the mobility resolved spectra.

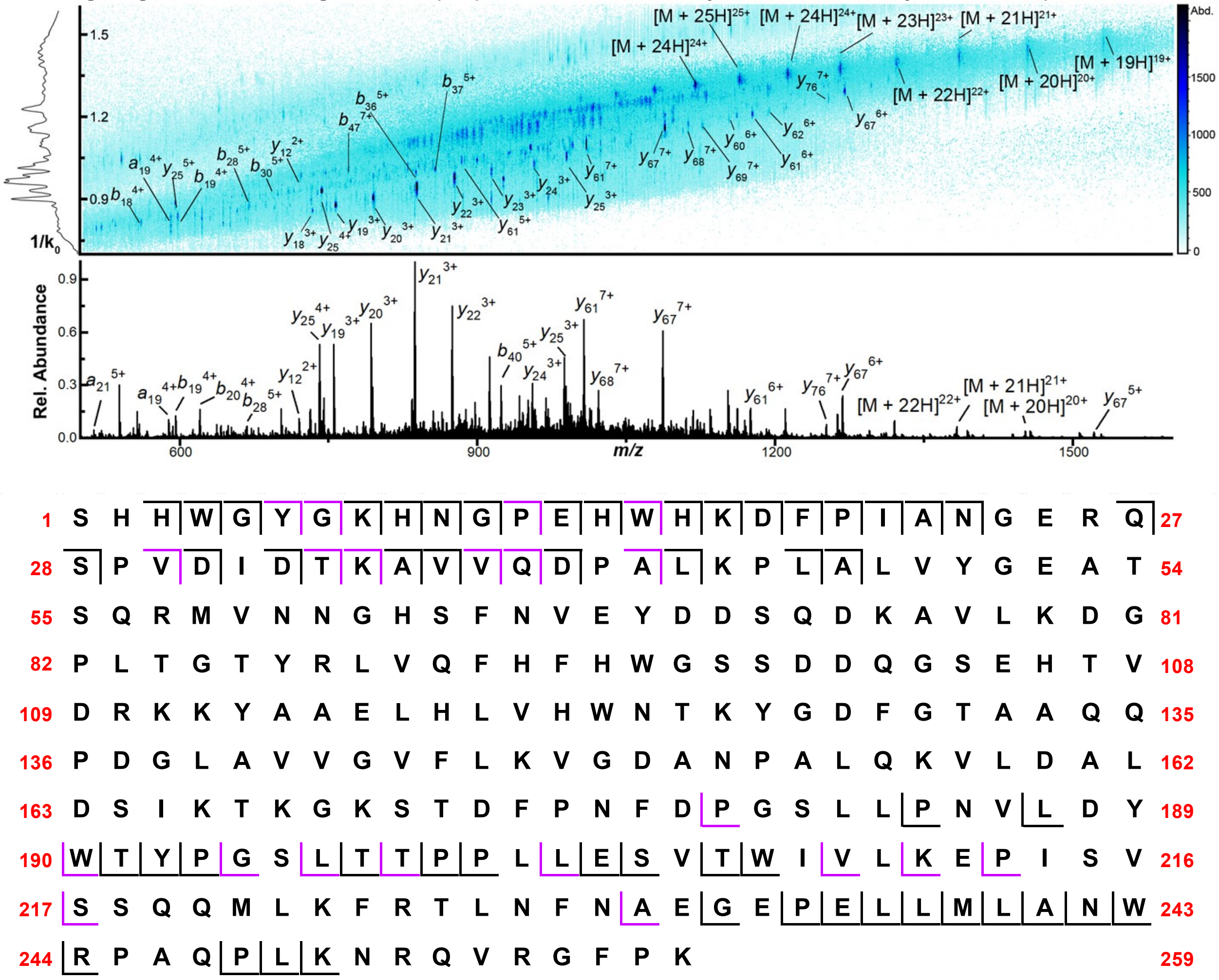

**Figure S10. BSA CIDtims spectrum, mobility spectrum, and 2D-IMS-MS**

Mass spectrum, mobility spectrum, and 2D-IMS-MS plot of 2 μM BSA generated by CIDtims when tunnel-in pressure is 1.5 mbar, accumulation time is 20 ms, and Δ6 is 150 V with ladder of resulting fragment ions. Fragments in purple are identified only from the mobility resolved spectra.

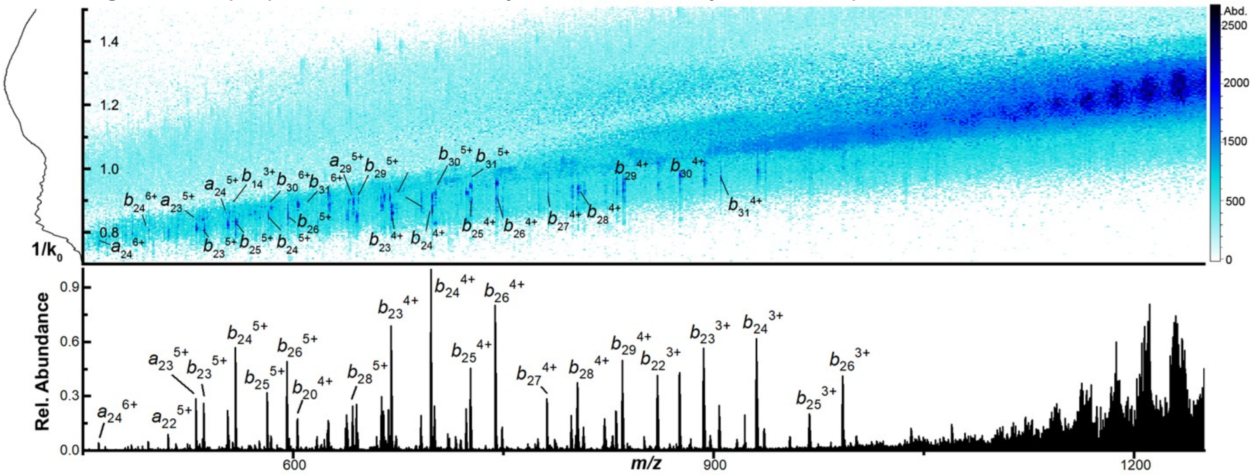


1 D T H K S E I A H R F K D L G E E H F K G L V L I A F 27

28 S Q Y L Q Q C P F D E H V K L V N E L T E F A K T C V 54

55 A D E S H A G C E K S L H T L F G D E L C K V A S L R 81

82 E T Y G D M A D C C E K Q E P E R N E C F L S H K D D 108

109 S P D L P K L K P D P N T L C D E F K A D E K K F W G 135

136 K Y L Y E I A R R H P Y F Y A P E L L Y Y A N K Y N G 162

163 V F Q E C C Q A E D K G A C L L P K I E T M R E K V L 189

190 R S S A R Q R L R C A S I Q K F G E R A L K A W S V A 216

217 R L S Q K F P K A E F V E V T K L V T D L T K V H K E 243

244 C C H G D L L E C A D D R A D L A K Y I C D N Q D T I 270

271 S S K L K E C C D K P L L E K S H C I A E V E K D A I 297

298 P E N L P P L T A D F A E D K D V C K N Y Q E A K D A 324

325 F L G S F L Y E Y S R R H P E Y A V S V L L R L A K E 351

352 Y E A T L E E C C A K D D P H A C Y S T V F D K L K H 378

379 L V D E P Q N L I K Q N C D Q F E K L G E Y G F Q N A 405

406 L I V R Y T R K V P Q V S T P T L V E V S R S L G K V 432

433 G T R C C T K P E S E R M P C T E D Y L S L I L N R L 459

460 C V L H E K T P V S E K V T K C C T E S L V N R R P C 486

487 F S A L T P D E T Y V P K A F D E K L F T F H A D I C 513

514 T L P D T E K Q I K K Q T A L V E L L K H K P K A T E 540

541 E Q L K T V M E N F V A F V D K C C A A D D K E A C F 567

568 A V E G P K L V V S T Q T A L A 583

**Figure S11. Carbonic anhydrase mass spectra at various concentrations**
Charge state distribution of intact carbonic anhydrase as concentration is lowered and accumulation time is held constant at 20 ms. Relative abundance of each highlighted charge state is displayed on each spectrum.

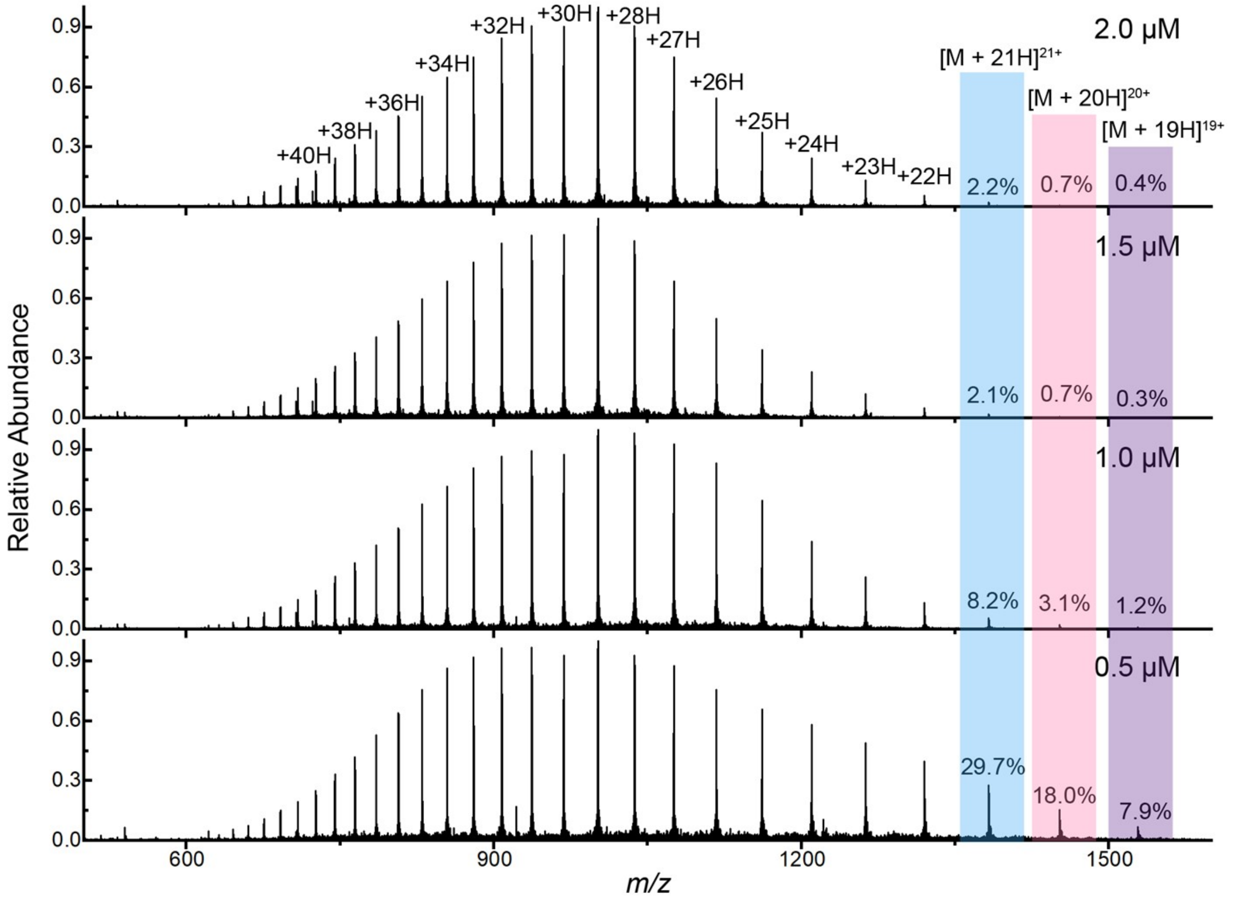

**Figure S12. Carbonic anhydrase CIDtims fragmentation ladder**

Resulting fragmentation ladder of CIDtims of carbonic anhydrase when accumulation time is 20 ms and concentration is 0.5 μM. Fragments in purple are identified only from the mobility resolved spectra.

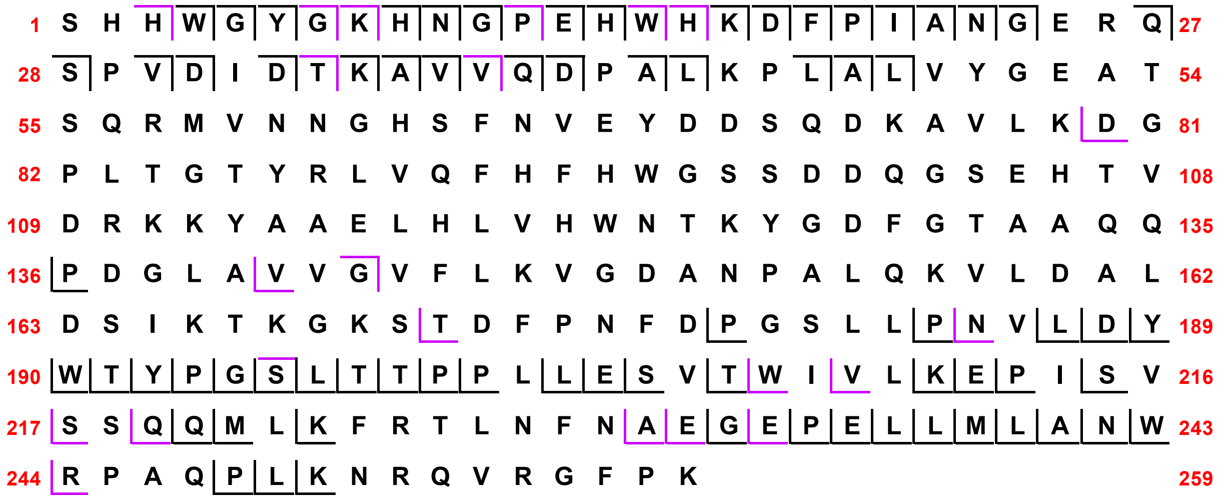

**Figure S13. Mass spectra of intact cytochrome C at indicated accumulation times**

Intact CytC distribution when accumulation is 100 ms (top) and 20 ms (bottom).

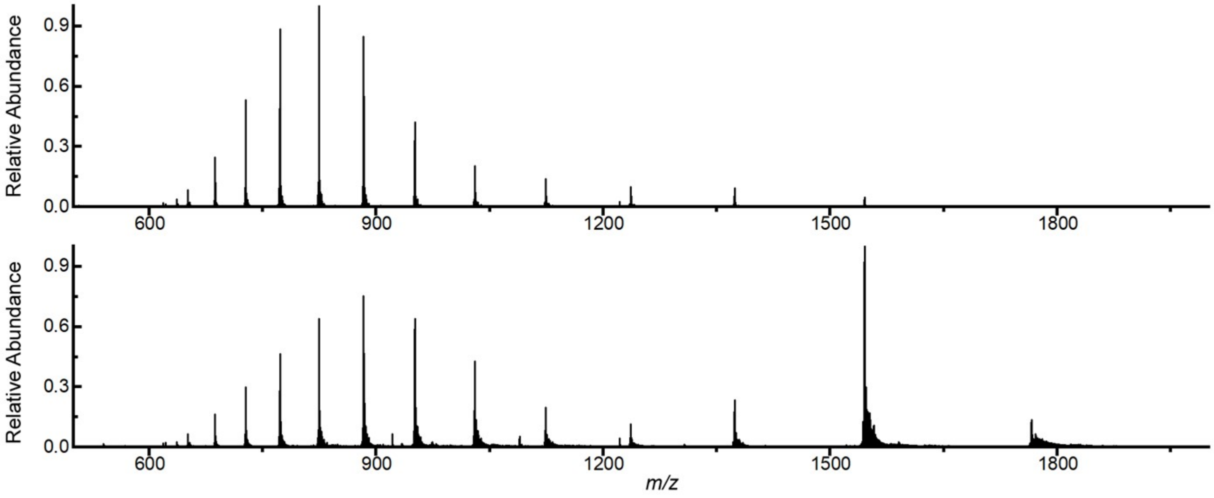


**Figure S14. Mass spectra of intact β-lactoglobulin at indicated accumulation times**

Intact BLG distribution when accumulation is 100 ms (top) and 20 ms (bottom).

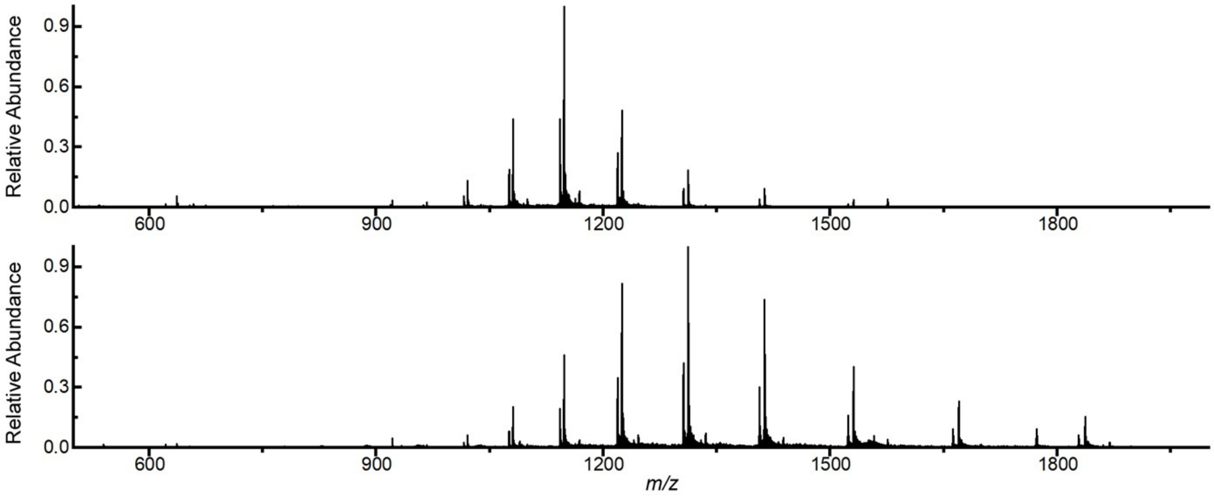

**Figure S15. Cytochrome C CIDtims spectrum, mobility spectrum, and 2D-IMS-MS with reduced accumulation**

Cytochrome C mass spectra, mobility resolved spectra, and 2D-IMS-MS when accumulation is 20 ms and Δ6=150 V with ladder of resulting fragment ions. Fragments in purple are identified only from the mobility resolved spectra.

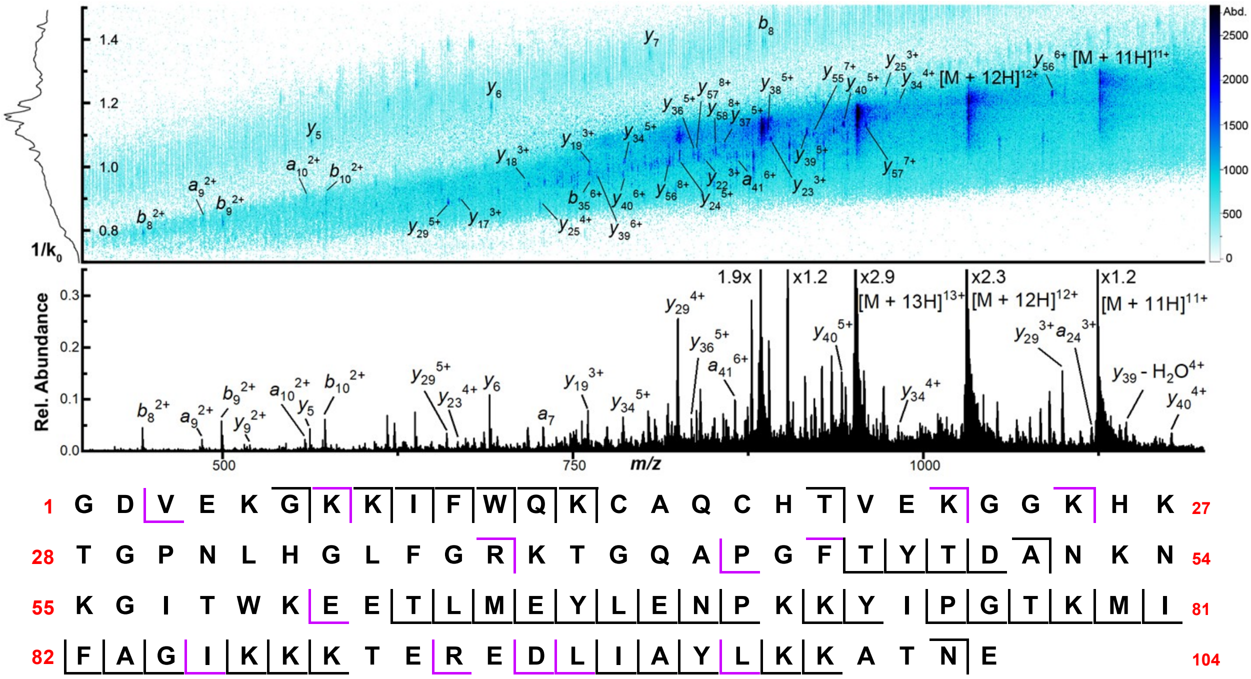

**Figure S16. β-lactoglobulin CIDtims spectrum, mobility spectrum, and 2D-IMS-MS with reduced accumulation**

β-lactoglobulin mass spectra, mobility resolved spectra, and 2D-IMS-MS when accumulation is 20 ms and Δ6=150 V with ladder of resulting fragment ions. Fragments in purple are identified only from the mobility resolved spectra.

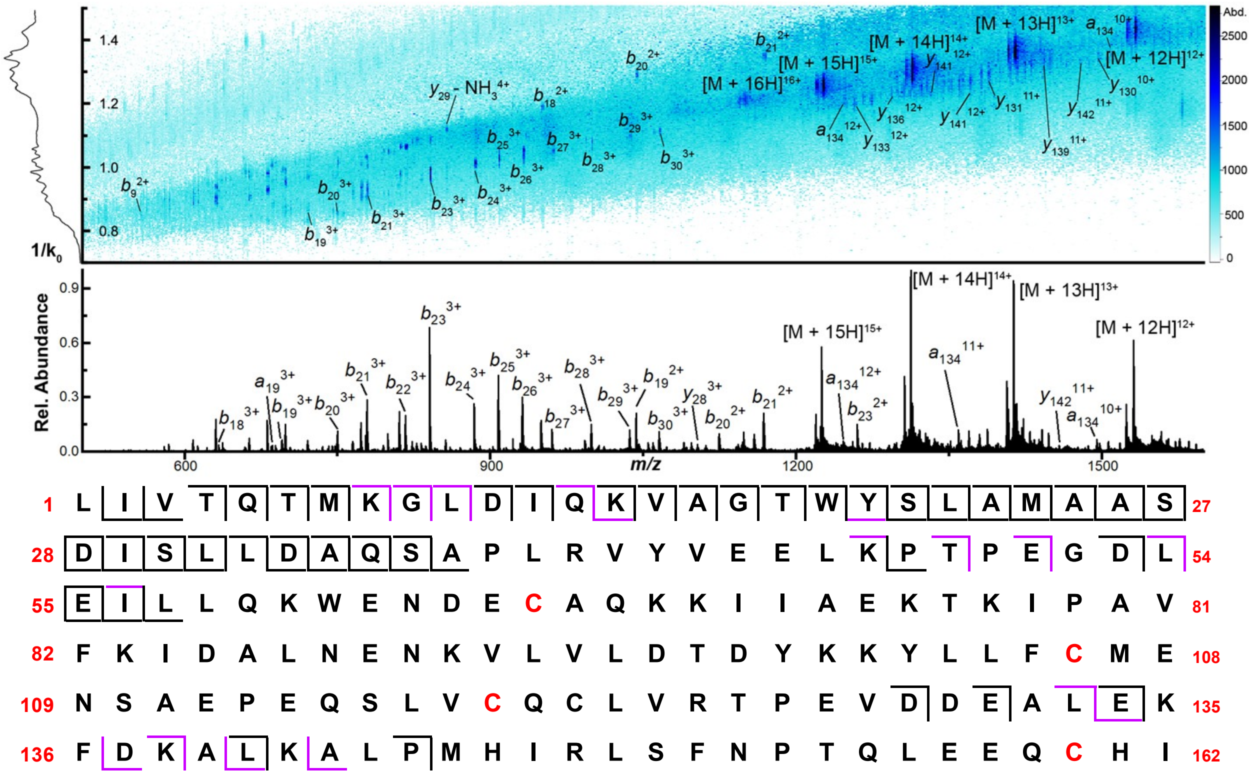


**Figure S17. Mass spectra of intact Ubiquitin at varied accumulation times**

Intact ubiquitin at 100 ms (Top) and 20 ms accumulation (Bottom).

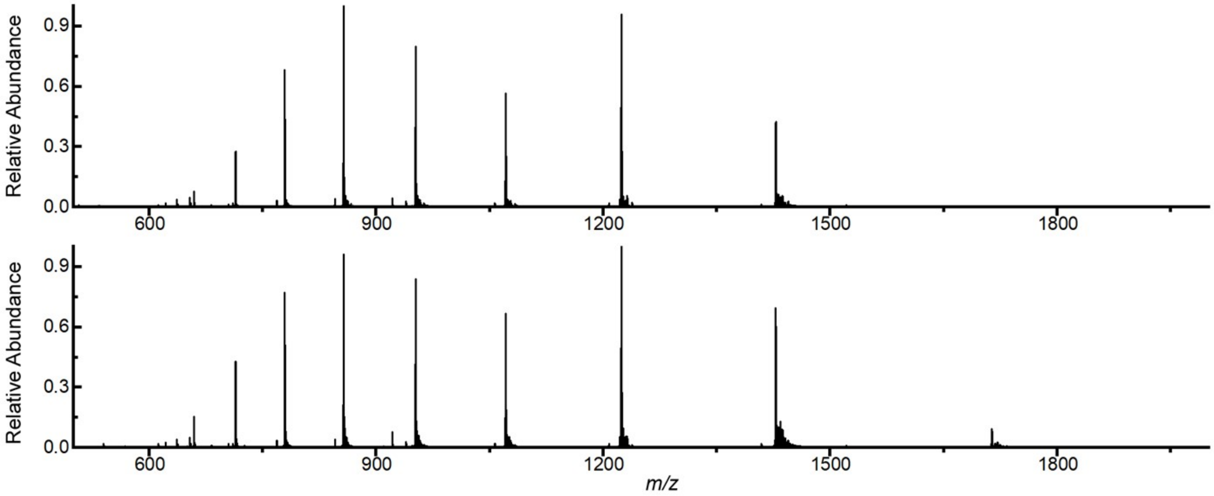

**Figure S18. Ubiquitin CIDtims mass spectrum, mobility spectrum, and 2D-IMS-MS with reduced accumulation**

Ubiquitin mass spectra, mobility resolved spectra, and 2D-IMS-MS when accumulation is 20 ms and Δ6=150 V with ladder of resulting fragment ions. Fragments in purple are identified only from the mobility resolved spectra.

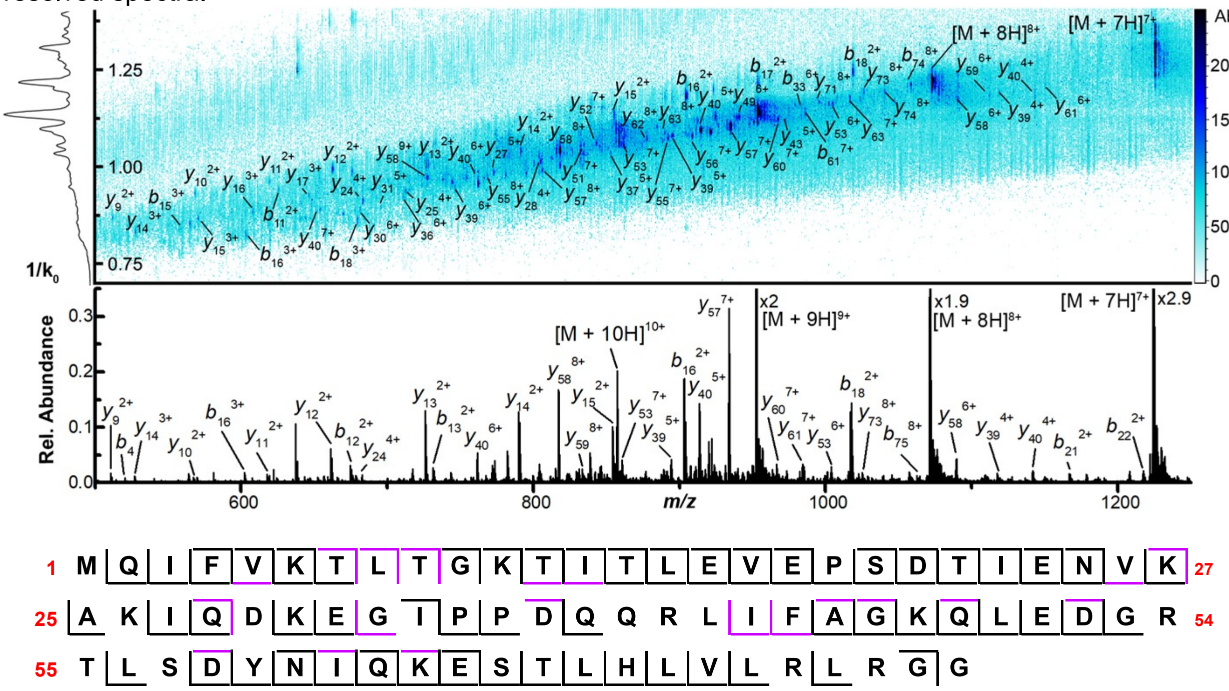

**Figure S19. BSA CIDtims spectrum, mobility spectrum, and 2D-IMS-MS at 0.5 μM**

BSA mass spectra, mobility resolved spectra, and 2D-IMS-MS when concentration is 0.5 μM, tunnel-in 1.5 mbar, 20 ms accumulation, and when Δ6=150 V with ladder of resulting fragment ions. Fragments in purple are identified only from the mobility resolved spectra.

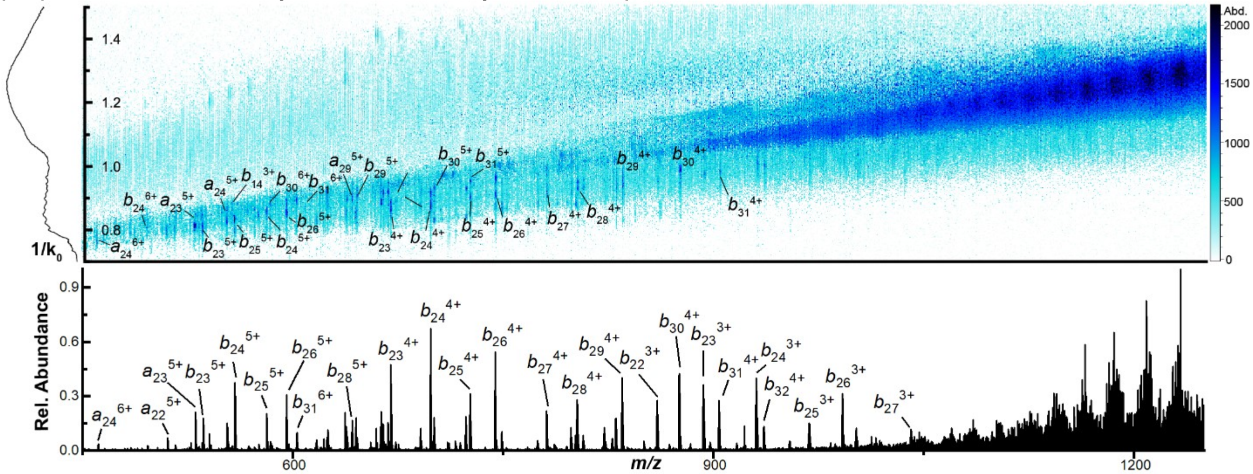


1 D T H K S E I A H R F K D L G E E H F K G L V L I A F 27
28 S Q Y L Q Q C P F D E H V K L V N E L T E F A K T C V 54
55 A D E S H A G C E K S L H T L F G D E L C K V A S L R 81
82 E T Y G D M A D C C E K Q E P E R N E C F L S H K D D 108
109 S P D L P K L K P D P N T L C D E F K A D E K K F W G 135
136 K Y L Y E I A R R H P Y F Y A P E L L Y Y A N K Y N G 162
163 V F Q E C C Q A E D K G A C L L P K I E T M R E K V L 189
190 R S S A R Q R L R C A S I Q K F G E R A L K A W S V A 216
217 R L S Q K F P K A E F V E V T K L V T D L T K V H K E 243
244 C C H G D L L E C A D D R A D L A K Y I C D N Q D T I 270
271 S S K L K E C C D K P L L E K S H C I A E V E K D A I 297
298 P E N L P P L T A D F A E D K D V C K N Y Q E A K D A 324
325 F L G S F L Y E Y S R R H P E Y A V S V L L R L A K E 351
352 Y E A T L E E C C A K D D P H A C Y S T V F D K L K H 378
379 L V D E P Q N L I K Q N C D Q F E K L G E Y G F Q N A 405
406 L I V R Y T R K V P Q V S T P T L V E V S R S L G K V 432
433 G T R C C T K P E S E R M P C T E D Y L S L I L N R L 459
460 C V L H E K T P V S E K V T K C C T E S L V N R R P C 486
487 F S A L T P D E T Y V P K A F D E K L F T F H A D I C 513
514 T L P D T E K Q I K K Q T A L V E L L K H K P K A T E 540
541 E Q L K T V M E N F V A F V D K C C A A D D K E A C F 567
568 A V E G P K L V V S T Q T A L A 583

**Figure S20. BSA CIDtims spectrum, mobility spectrum, and 2D-IMS-MS with ICC**

BSA mass spectra, mobility resolved spectra, and 2D-IMS-MS when concentration is 2 μM, tunnel-in 1.5 mbar, ICC is 3.5 mio, and when Δ6=150 V with ladder of resulting fragment ions. Fragments in purple are identified only from the mobility resolved spectra.

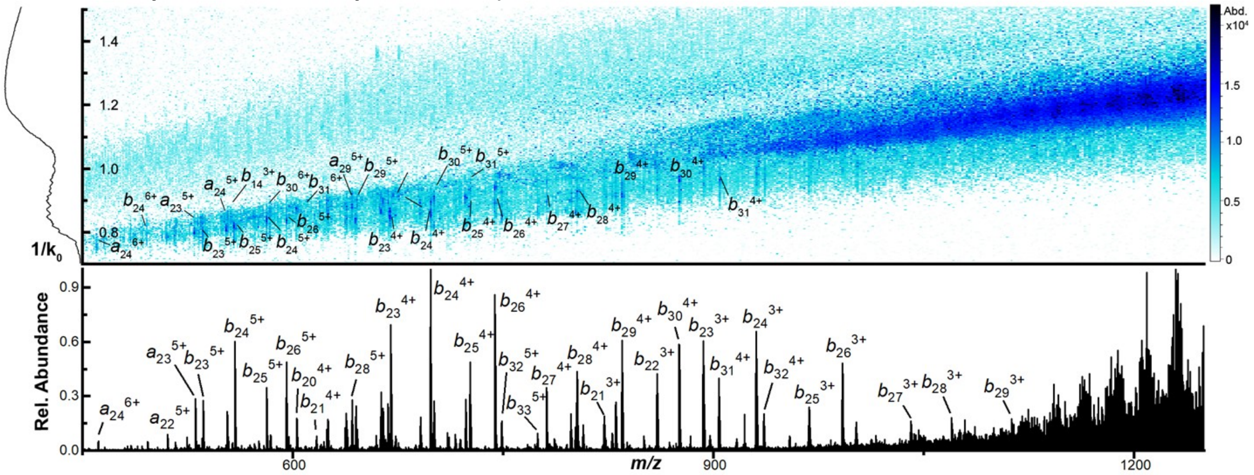


1 D T H K S E I A H R F K D L G E E H F K G L V L I A F 27
28 S Q Y L Q Q C P F D E H V K L V N E L T E F A K T C V 54
55 A D E S H A G C E K S L H T L F G D E L C K V A S L R 81
82 E T Y G D M A D C C E K Q E P E R N E C F L S H K D D 108
109 S P D L P K L K P D P N T L C D E F K A D E K K F W G 135
136 K Y L Y E I A R R H P Y F Y A P E L L Y Y A N K Y N G 162
163 V F Q E C C Q A E D K G A C L L P K I E T M R E K V L 189
190 R S S A R Q R L R C A S I Q K F G E R A L K A W S V A 216
217 R L S Q K F P K A E F V E V T K L V T D L T K V H K E 243
244 C C H G D L L E C A D D R A D L A K Y I C D N Q D T I 270
271 S S K L K E C C D K P L L E K S H C I A E V E K D A I 297
298 P E N L P P L T A D F A E D K D V C K N Y Q E A K D A 324
325 F L G S F L Y E Y S R R H P E Y A V S V L L R L A K E 351
352 Y E A T L E E C C A K D D P H A C Y S T V F D K L K H 378
379 L V D E P Q N L I K Q N C D Q F E K L G E Y G F Q N A 405
406 L I V R Y T R K V P Q V S T P T L V E V S R S L G K V 432
433 G T R C C T K P E S E R M P C T E D Y L S L I L N R L 459
460 C V L H E K T P V S E K V T K C C T E S L V N R R P C 486
487 F S A L T P D E T Y V P K A F D E K L F T F H A D I C 513
514 T L P D T E K Q I K K Q T A L V E L L K H K P K A T E 540
541 E Q L K T V M E N F V A F V D K C C A A D D K E A C F 567
568 A V E G P K L V V S T Q T A L A 583

**Figure S21. Ubiquitin isCID mass spectrum and ladder**

Spectrum and resulting fragment ladder of ubiquitin after activation by isCID.

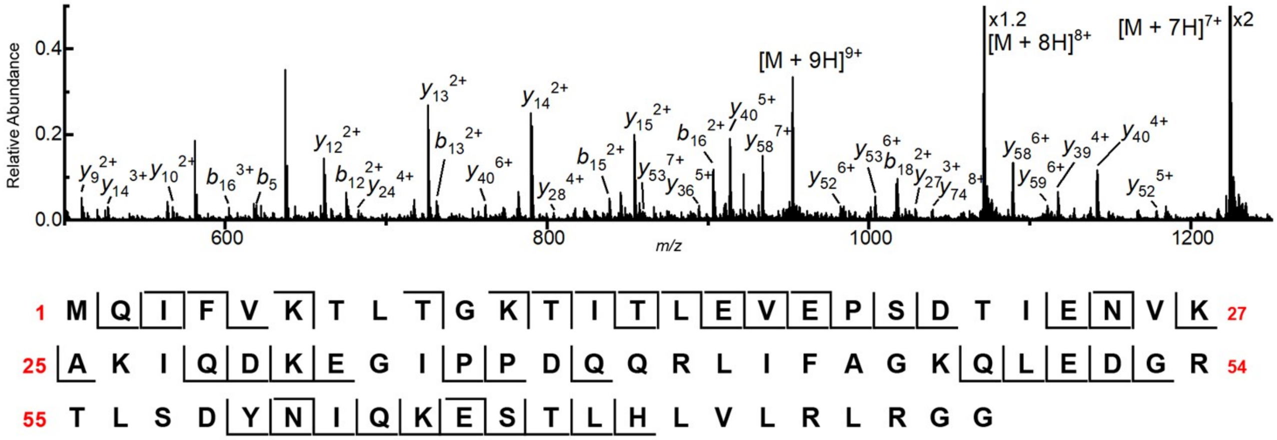


**Figure S22. Cytochrome C isCID mass spectrum and ladder**

Spectrum and resulting fragment ladder of cytochrome C after activation by isCID.

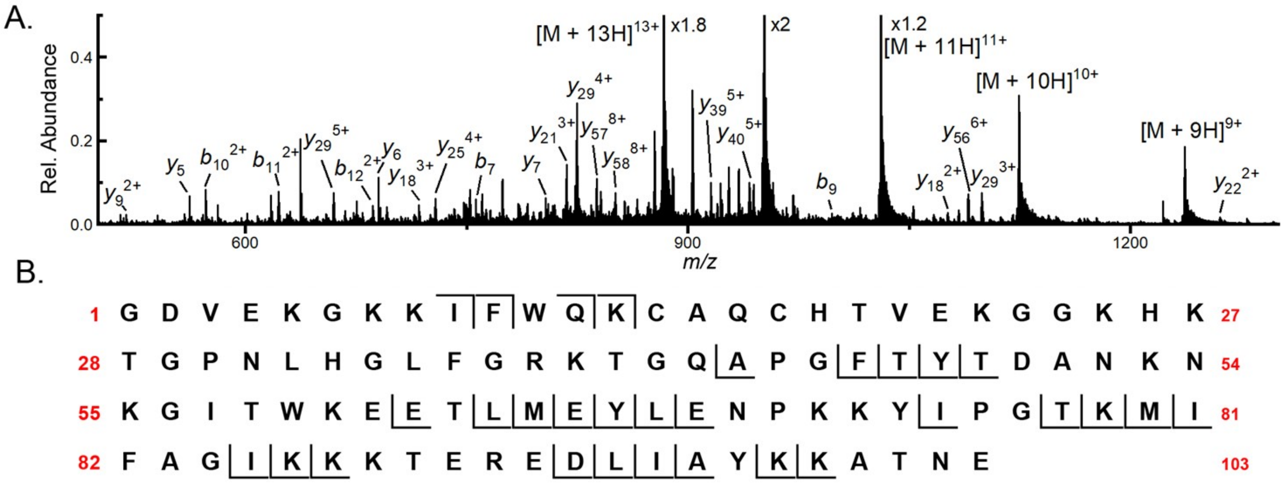

**Figure S23. β-lactoglobulin isCID mass spectrum and ladder**

Spectrum and resulting fragment ladder of β-lactoglobulin after activation by isCID.

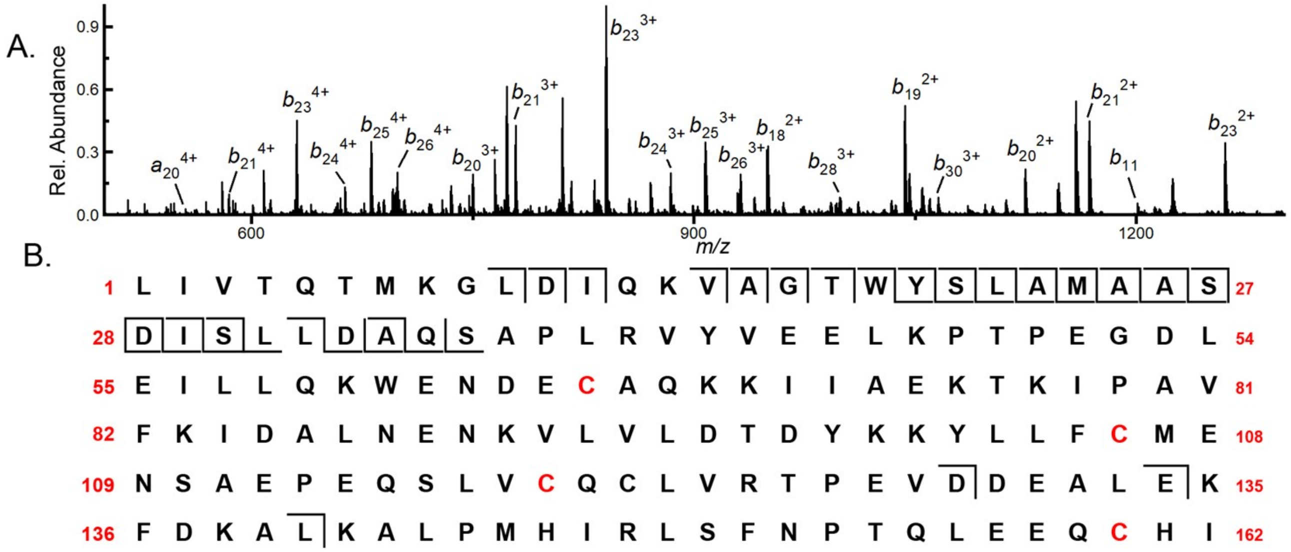

**Figure S24. Carbonic Anhydrase isCID mass spectrum and ladder**

Spectrum and resulting fragment ladder of carbonic anhydrase after activation by isCID.

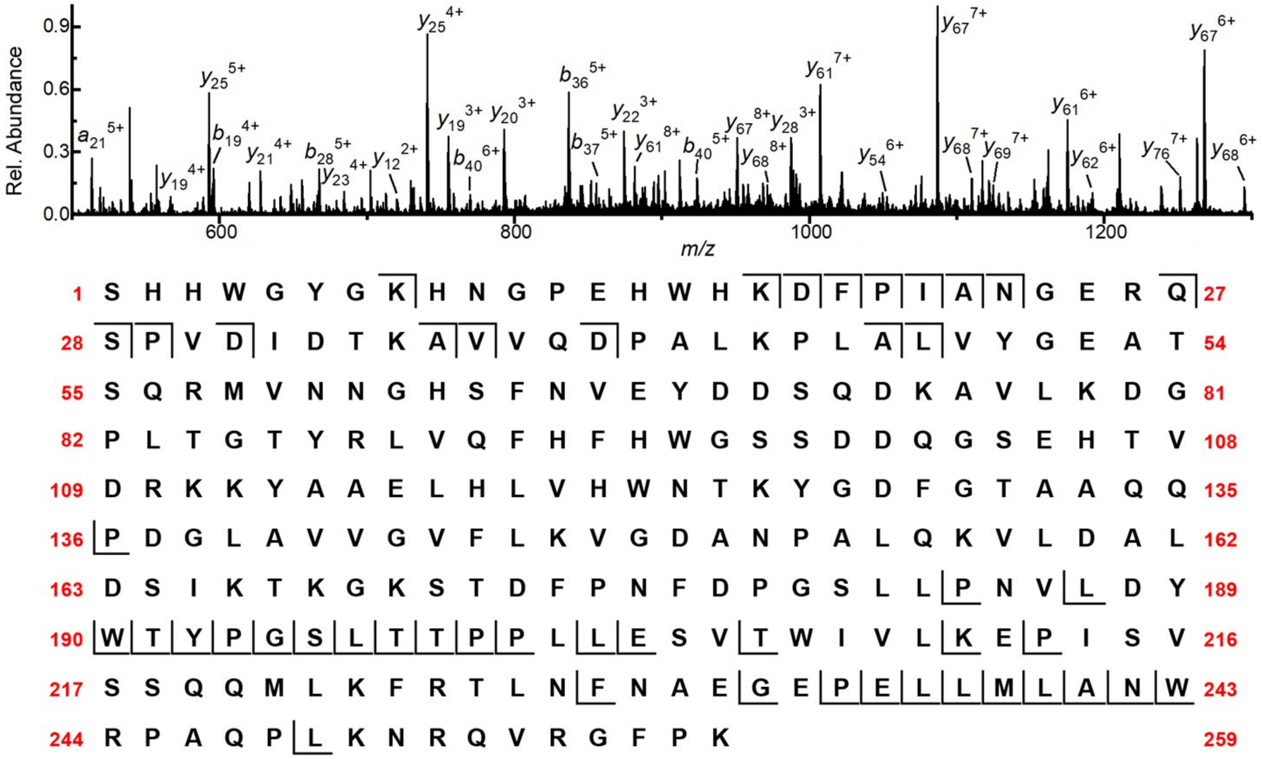

**Figure S25. BSA isCID mass spectrum and ladder**

Spectrum and resulting fragment ladder of BSA after activation by isCID.

A.

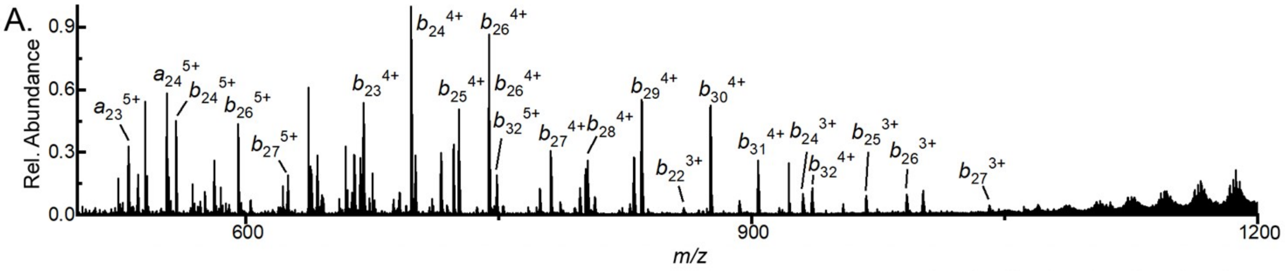


B.

1 D T H K S E I A H R F K D L G E E H F K G L V L I A F 27
28 S Q Y L Q Q C P F D E H V K L V N E L T E F A K T C V 54
55 A D E S H A G C E K S L H T L F G D E L C K V A S L R 81
82 E T Y G D M A D C C E K Q E P E R N E C F L S H K D D 108
109 S P D L P K L K P D P N T L C D E F K A D E K K F W G 135
136 K Y L Y E I A R R H P Y F Y A P E L L Y Y A N K Y N G 162
163 V F Q E C C Q A E D K G A C L L P K I E T M R E K V L 189
190 R S S A R Q R L R C A S I Q K F G E R A L K A W S V A 216
217 R L S Q K F P K A E F V E V T K L V T D L T K V H K E 243
244 C C H G D L L E C A D D R A D L A K Y I C D N Q D T I 270
271 S S K L K E C C D K P L L E K S H C I A E V E K D A I 297
298 P E N L P P L T A D F A E D K D V C K N Y Q E A K D A 324
325 F L G S F L Y E Y S R R H P E Y A V S V L L R L A K E 351
352 Y E A T L E E C C A K D D P H A C Y S T V F D K L K H 378
379 L V D E P Q N L I K Q N C D Q F E K L G E Y G F Q N A 405
406 L I V R Y T R K V P Q V S T P T L V E V S R S L G K V 432
433 G T R C C T K P E S E R M P C T E D Y L S L I L N R L 459
460 C V L H E K T P V S E K V T K C C T E S L V N R R P C 486
487 F S A L T P D E T Y V P K A F D E K L F T F H A D I C 513
514 T L P D T E K Q I K K Q T A L V E L L K H K P K A T E 540
541 E Q L K T V M E N F V A F V D K C C A A D D K E A C F 567
568 A V E G P K L V V S T Q T A L A 583

**Figure S26. Ubiquitin CID spectrum and ladder**

A. CID tandem mass spectrum of 12+ charge state of Ubiquitin. B. Ladder indicates sequence information provided by pooling the product ions generated by dissociating the 10+, 11+, and 12+ charge states of ubiquitin.

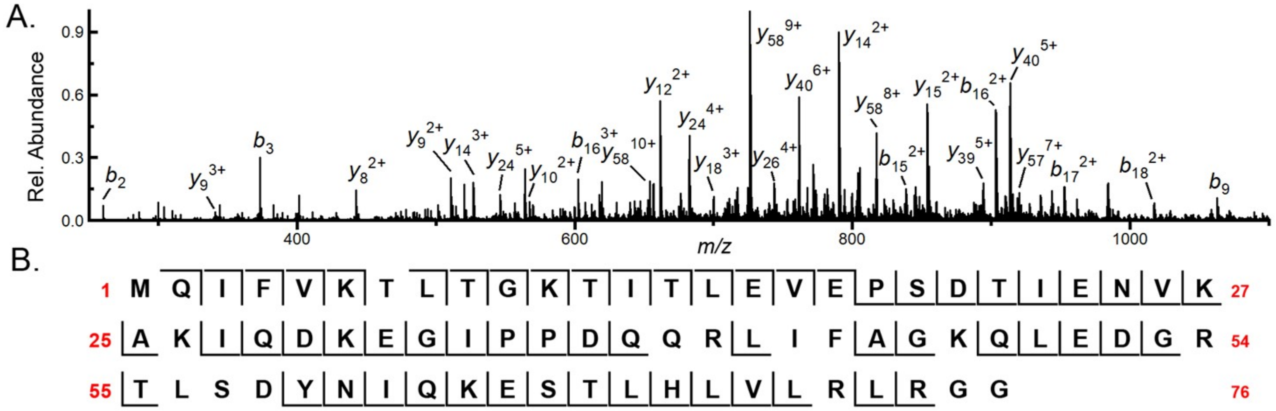


**Figure S27. Venn diagram of CID-generated product ions for indicated charge states.**

Venn diagrams indicate the number of unique and shared sequence informative product ions generated from dissociation of each indicated charge state for the proteins **A.** ubiquitin **B.** cytochrome C **C.** β-lactoglobulin **D.** carbonic anhydrase and **E.** BSA

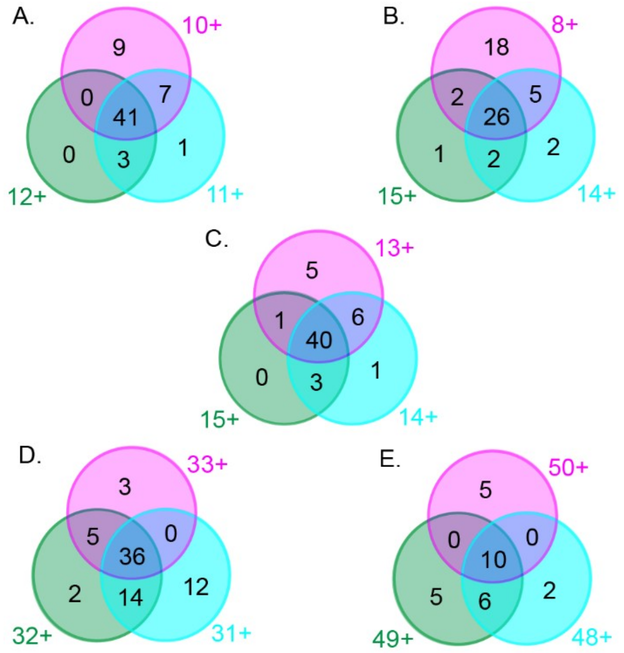

**Figure S28. Tandem mass spectrum of cytochrome C following CID**

A. CID tandem mass spectrum of 15+ charge state of Cytochrome C. B. Ladder indicates sequence information provided by pooling the product ions generated by dissociating the 8+, 14+, and 15+ charge states of cytochrome C.

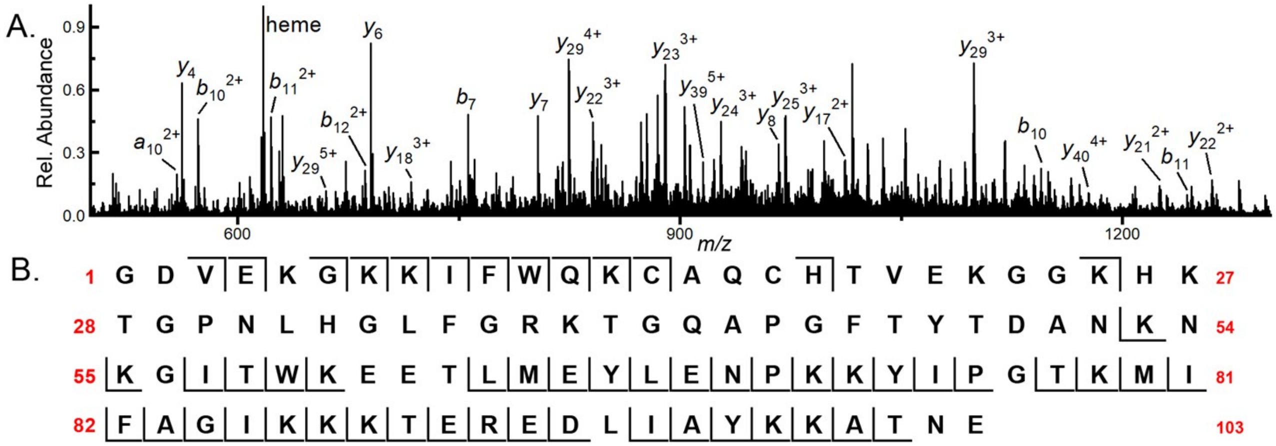


**Figure S29. Tandem mass spectrum of β-lactoglobulin following CID**

A. CID tandem mass spectrum of 15+ charge state of β-lactoglobulin. B. Ladder indicates sequence information provided by pooling the product ions generated by dissociating the 13+, 14+, and 15+ charge states of β-lactoglobulin.

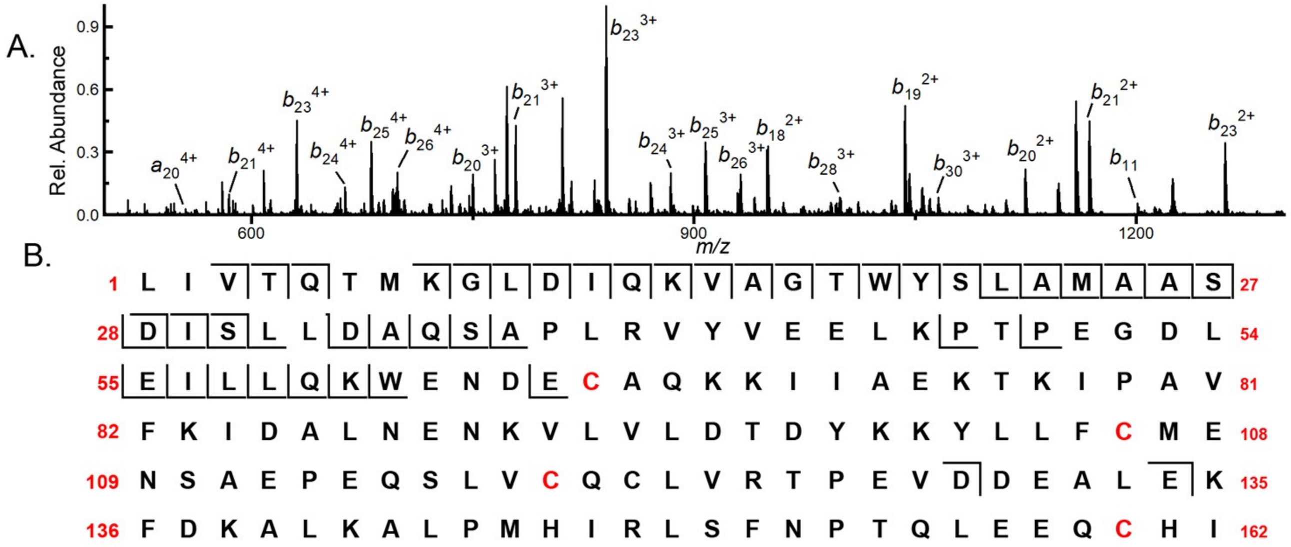

**Figure S30. Tandem mass spectrum of Carbonic anhydrase following CID**
A. CID tandem mass spectrum of 33+ charge state of carbonic anhydrase. B. Ladder indicates sequence information provided by pooling the product ions generated by dissociating the 31+, 32+, and 33+ charge states of carbonic anhydrase.

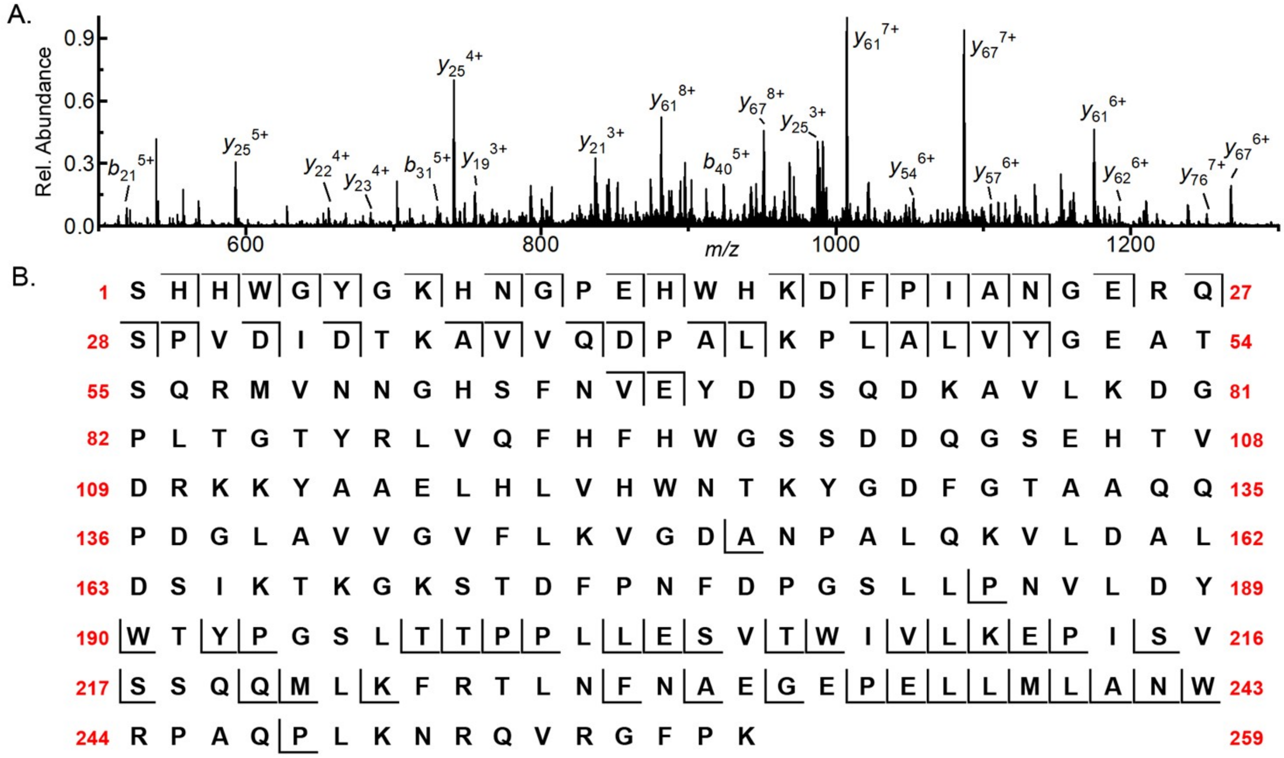

**Figure S31. Tandem mass spectrum of BSA following CID**
A. CID tandem mass spectrum of 50+ charge state of BSA. B. Ladder indicates sequence information provided by pooling the product ions generated by dissociating the 48+, 49+, and 50+ charge states of BSA.

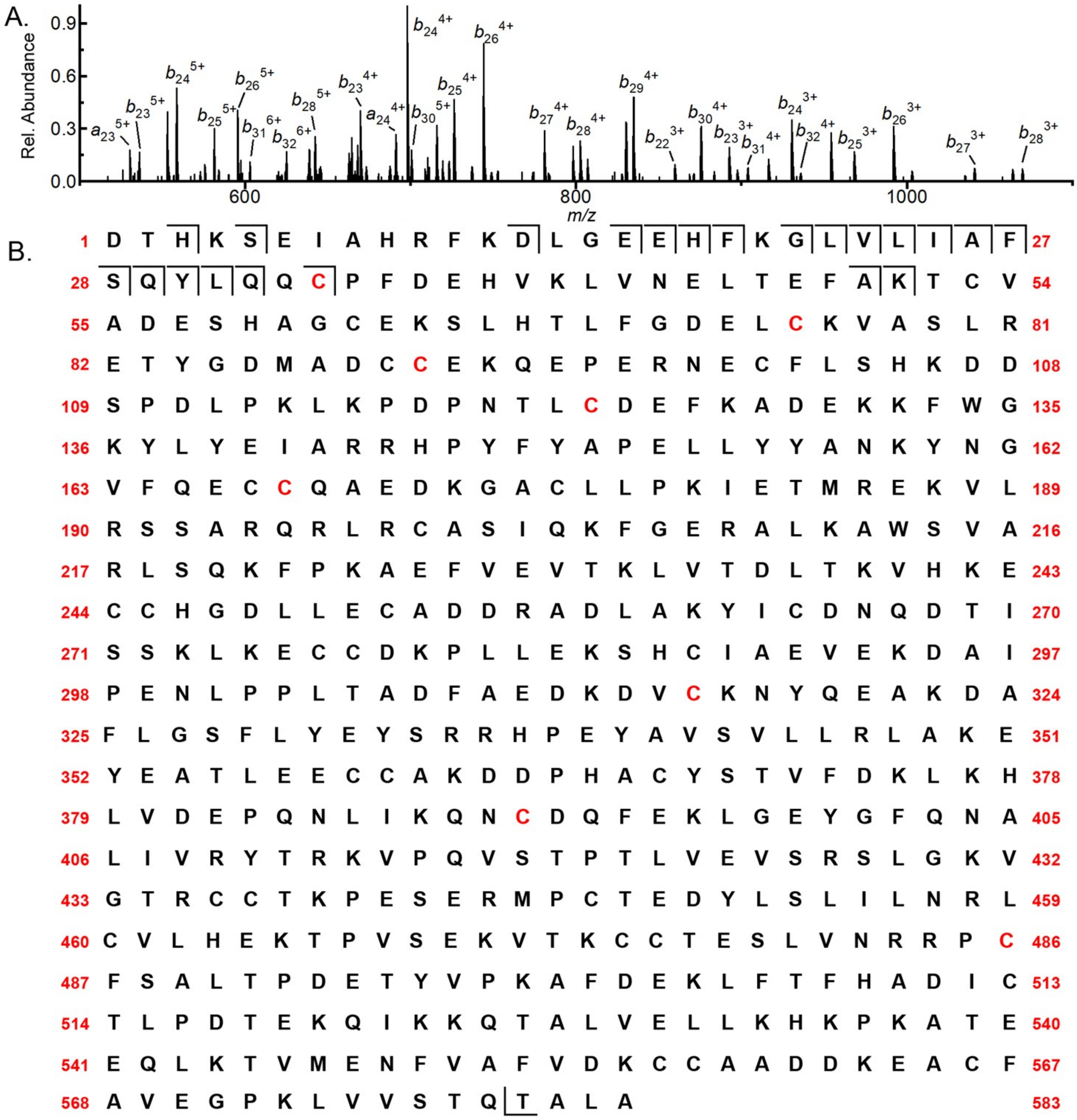

Supplement: AN-148-D2AN01682F-s001 [file AN-148-D2AN01682F-s001.pdf]
